# A comprehensive RNA handling and transcriptomics guide for high-throughput processing of *Plasmodium* blood-stage samples

**DOI:** 10.1186/s12936-020-03436-w

**Published:** 2020-10-09

**Authors:** Michal Kucharski, Jaishree Tripathi, Sourav Nayak, Lei Zhu, Grennady Wirjanata, Rob W. van der Pluijm, Mehul Dhorda, Arjen Dondorp, Zbynek Bozdech

**Affiliations:** 1 School of Biological Sciences, Nanyang Technological University, Singapore, 637551 Singapore; 2 Mahidol Oxford Tropical Medicine Research Unit, Faculty of Tropical Medicine, Mahidol University, Bangkok, Thailand; 3 Centre for Tropical Medicine and Global Health, Nuffield Department of Medicine, University of Oxford, Oxford, UK; 4 WorldWide Antimalarial Resistance Network–Asia Regional Centre, Bangkok, Thailand

**Keywords:** Whole transcriptome analysis, RNA extraction, RNA preservation, *Plasmodium*, RNA-seq, High-throughput, Malaria

## Abstract

**Background:**

Sequencing technology advancements opened new opportunities to use transcriptomics for studying malaria pathology and epidemiology. Even though in recent years the study of whole parasite transcriptome proved to be essential in understanding parasite biology there is no compiled up-to-date reference protocol for the efficient generation of transcriptome data from growing number of samples. Here, a comprehensive methodology on how to preserve, extract, amplify, and sequence full-length mRNA transcripts from *Plasmodium*-infected blood samples is presented that can be fully streamlined for high-throughput studies.

**Results:**

The utility of various commercially available RNA-preserving reagents in a range of storage conditions was evaluated. Similarly, several RNA extraction protocols were compared and the one most suitable method for the extraction of high-quality total RNA from low-parasitaemia and low-volume blood samples was established. Furthermore, the criteria needed to evaluate the quality and integrity of *Plasmodium* RNA in the presence of human RNA was updated. Optimization of SMART-seq2 amplification method to better suit AT-rich *Plasmodium falciparum* RNA samples allowed us to generate high-quality transcriptomes from as little as 10 ng of total RNA and a lower parasitaemia limit of 0.05%. Finally, a modified method for depletion of unwanted human haemoglobin transcripts using in vitro CRISPR-Cas9 treatment was designed, thus improving parasite transcriptome coverage in low parasitaemia samples. To prove the functionality of the pipeline for both laboratory and field strains, the highest  2-hour resolution RNA-seq transcriptome for *P. falciparum* 3D7 intraerythrocytic life cycle available to  date was generated, and the entire protocol was applied to create the largest transcriptome data from Southeast Asian field isolates.

**Conclusions:**

Overall, the presented methodology is an inclusive pipeline for generation of good quality transcriptomic data from a diverse range of *Plasmodium*-infected blood samples with varying parasitaemia and RNA inputs. The flexibility of this pipeline to be adapted to robotic handling will facilitate both small and large-scale future transcriptomic studies in the field of malaria.

## Background

Malaria still accounts for 405,000 deaths worldwide [1]. Infection in humans is caused by at least five different species of *Plasmodium* parasites, with *Plasmodium falciparum* causing the highest mortality globally. Despite numerous anti-malarial medications being used for treatment, the current frontline anti-malarial treatment – artemisinin-based combination therapy, is starting to fail in several regions of Southeast Asia due to the emergence of drug resistance [2, 3]. This raises serious concerns towards malaria eradication. All this necessitates a better understanding of molecular mechanisms underlying malaria pathogenesis, parasite virulence, transmission, and drug resistance, eventually contributing to the development of new chemotherapeutics and vaccine strategies to combat malaria.

Clinical manifestations of malaria result from the asexual stage of the parasite multiplying during the intraerythrocytic development cycle (IDC). Both, parasite load and host inflammatory response are an indicator of disease severity [4, 5]. Molecular interactions between host and parasite occurring within blood vessels and other organs, such as the spleen, have been shown to be associated with the development of malaria clinical symptoms. For instance, *var* gene family encoding for clonally variant surface antigens, *P. falciparum* erythrocyte membrane protein 1 (PfEMP1), has been shown to interact with endothelial protein C receptor or chondroitin sulphate A resulting in cytoadherence of infected red blood cells (iRBCs) in host blood vessels and placenta, respectively [6, 7]. This results in symptomatic complications, such as, development of severe malaria and pregnancy-associated malaria. RIFINs and STEVORs on the other hand have also been implicated as adhesins facilitating rosetting and merozoite invasion.

Recent advances in molecular biology techniques and bioinformatics tools have enabled studying RNA expression at the whole-genome level like never before. The first high resolution *P. falciparum* blood-stage transcriptome was published in 2003, where a “just-in-time” model for transcription leading to a continuous cascade of gene expression was proposed [8]. This was followed by several other studies where transcriptome analyses of malaria blood stages demonstrated how variation in gene expression is tightly linked to achieving diverse parasite phenotypes, adaptation, immune evasion, reproduction, and transmission. For example, gene expression studies on sexual commitment have revealed the master regulator ApiAP2-G which regulates transcriptional activation of several gametocyte differentiation genes, thus converting late-stage asexual parasites into gametocytes [9].

Understanding drug resistance mechanisms is another area of research that has invaluably benefited from transcriptional profiling of malaria clinical isolates. Studies on in vivo transcriptomes of *P. falciparum* isolates from South East Asia have demonstrated that artemisinin drug resistance is associated with distinct transcriptional signature characterized by increased expression of unfolded protein response involving PROSC and TRiC chaperone complexes. In a separate study analysing the same dataset, transcriptomic and genetic backgrounds that predispose *P. falciparum* to acquire artemisinin resistance were reported [10, 11]. Moreover, two other studies have conducted whole-transcriptome profiling of severe and acute malaria isolates, aiming to reveal mechanisms driving the systemic pathophysiology of severe malaria and parasite factors that induce apoptosis of human endothelial cells resulting in the disruption of the blood–brain barrier, respectively [12, 13]. Overall, these research studies indicate growing popularity of applying the whole-transcriptome profiling approach to achieve a holistic view of the biological mechanisms in action during malaria infection.

The aim of this study is to provide a comprehensive methodology on how to store, extract, process, and analyse parasite RNA from *P. falciparum*-infected blood samples. One of the key features of the presented pipeline is that it is easily adaptable to low and high-throughput processing of malaria blood samples with either microarray or RNA-sequencing (RNA-seq) being used as a readout platform. Furthermore, a lower threshold of 0.05% parasitaemia (approximately 2000–3000 parasites/µl of whole blood), 8 µl iRBCs volume, and 10 ng total RNA input were independently established as necessary to generate satisfactory transcriptome coverage.

The first step to generate a good quality malaria transcriptome requires isolation of high-quality RNA. Malaria clinical samples collected from remote field sites pose the challenge of proper long-term preservation during transportation to research labs for further processing. To address this, a systematic testing of various temperatures (4, 21, 37 °C), storage times (days to weeks), and preserving media (DNA/RNA Shield™ and TRIzol™) suitable for RNA preservation was conducted. Furthermore, several commercially available RNA isolation kits tested  were unable to process malaria blood samples. Thus, an optimized RNA isolation method suitable for both laboratory cultures and clinical malaria samples is discussed here. Likewise, to address the hurdles of low parasitaemia and low amounts of total RNA available for transcriptome analyses of clinical malaria isolates, the presented RNA processing pipeline can be applied to a range of parasitaemia (0.001% to 1%) and total RNA inputs (10–250 ng), demonstrating its utility for a wide variety of malaria blood samples. Lastly, the SMART-seq2 amplification method was optimized to better suit highly AT-rich *P. falciparum* RNA and an optional CRISPR-Cas9 mediated in vitro depletion of haemoglobin transcripts by up to 70% in low parasitaemia samples was introduced, thus improving transcriptome coverage. Overall, this methodology presents a comprehensive pipeline for generating good quality transcriptomic data from a diverse range of *Plasmodium*-infected blood samples.

## Methods

### Parasite culture

*Plasmodium falciparum* 3D7 strain was maintained in purified human packed red blood cells (RBCs) in RPMI 1640 medium (Gibco) supplemented with Albumax I (Gibco) (0.25%), hypoxanthine (Sigma) (0.1 mM), sodium bicarbonate (Sigma) (2 g/L), and gentamicin (Gibco) (50 μg/L). Cultures were kept at 37 °C with 5% CO_2_, 3% O_2_, and 92% N_2_. Culture media were replenished every 24 h with freshly washed packed RBCs added to the culture when necessary. Both parasitaemia and parasite morphology were assessed by microscopic examination of blood smears stained with Giemsa (Sigma).

### Intraerythrocytic asexual developmental cycle reference time course

*Plasmodium falciparum* strain 3D7 was obtained from BEI Resources (MRA-102) and cultured under the conditions described above. Prior to time-point experiment, parasites were double-synchronized with 5% sorbitol solution to achieve a synchrony of ± 6 h and cultured under constant agitation [14]. For sampling of highly synchronous parasites during the asexual life cycle, the first time point was considered as the TP1 (Time Point 1) when > 95% of early ring stage parasites (approx. 4 hours post invasion (hpi)) were present in the culture. To ensure a sufficient amount of mRNA for subsequent analysis, parasites were cultured in 25 individual flasks in 25 mL of medium at 2% haematocrit and 8% parasitaemia for each time point. Starting from TP1, parasites were collected every 2 h for 25 successive time points. Parasite development was monitored by Giemsa staining and media change was performed once at 24 h time point to maintain parasite growth. The 9th time point was removed due to its big dissimilarity to all the other samples, and the total of 24 time points were used here to build an asexual reference transcriptome generated using two different platforms: microarrays and RNA-seq.

### RNA processing

All RNA preservation, extraction, amplification, depletion and sequencing analyses are described in detail in Additional file 1: Supplementary protocol.

### RNA/DNA quality control

All RNA samples were analysed for integrity using Agilent Bioanalyzer 2100 platform. RNA 6000 Nano Chip (Cat. No.: 5067-1511, Agilent) was used for RNA samples according to manufacture’s instructions. For 18S-Pf/18S-Hs peak ratio analysis, only samples with both peaks present (human and parasite) were considered and samples with RNA Integrity Number (RIN) below 5 were excluded. Complementary DNA (cDNA) samples were assessed using DNA 12000 Kit (Cat. No.: 5067-1509, Agilent) according to manufacturer’s instructions.

### RNA-sequencing

Purified cDNA was used to generate sequencing libraries using Illumina Nextera XT kit as described in manufacturer’s protocol. Purified cDNA libraries were analysed on the Bioanalyzer High-Sensitivity DNA chips (Cat. No.: 5067-4626, Agilent), subsequently pooled (20–24 samples per lane) and sequenced on Illumina HiSeq4000 platform generating 150 bp paired end reads with 110 Gb data output generated per lane.

### Microarray hybridization

Previously described microarray hybridization protocol was used for this study with several modifications [15]. In brief, 100 ng of cDNA was used for subsequent 10 rounds of amplification to generate aminoallyl-coupled cDNA for the hybridizations as described [15]. 17ul (~ 5 µg) of each Cy-5-labelled (GE Healthcare) cDNA of the sample and an equal amount of Cy-3-labelled (GE Healthcare) cDNA of the reference pool were then hybridized together on customized microarray chip using commercially available hybridization platform (Agilent) for 20 h at 70 °C with rotation at 10 rpm. Microarrays were washed and immediately scanned using Power Scanner (Tecan) at 10 µm resolution and with automated photomultiplier tubes gain adjustments to balance the signal intensities between both channels. The reference pool used for microarray was a mixture of 3D7 parasite strain RNA collected every 6 h during 48 h of the full IDC.

### Data analysis

For microarray data signal intensities were Loess-normalized within arrays followed by quantile-normalization between samples/arrays using Limma package of R [16]. Missing values were assigned to probes with signal showing median foreground intensity less than 1.5-fold of the median background intensity at either Cy5 (sample RNA) or Cy3 (reference pool RNA) channel. Each gene expression was estimated as the average of log2 ratios (Cy5/Cy3) of representative probes. All scripts used for normalization and probe averaging are available from authors upon request.

Detailed step-by-step guide for RNA-seq data analysis can be found in the Additional file 1: Supplementary Protocol.

## Results

Numerous studies conducted in the last decade showed the potential of utilizing peripheral blood samples from malaria-infected patients to enable studies of in vivo gene expression profiles of malaria parasites including the deadliest species, *P. falciparum *[10, 11, 13, 15]. For that, it was essential to develop methods for optimal extraction, amplification, and detection of RNA in these samples. Here, a compilation of all methodological aspects for studies of in vivo transcriptomes of *P. falciparum* is presented and supported by extensive laboratory-based optimizations in order to provide a comprehensive methodological “manual” for conducting transcriptomic studies of malaria parasites from clinical samples. The results supporting this pipeline come from optimizations using in vitro laboratory cultures of *P. falciparum* (3D7) and a large set of clinical samples (n = 710) (data not shown) from patients with uncomplicated malaria infections collected across Greater Mekong Subregion during the Tracking Resistance to Artemisinin Collaboration study (i.e. TRAC2) conducted between 2016 and 2018 [17].

### RNA preservation, extraction and quantification of malaria blood samples

**Fig. 1:**
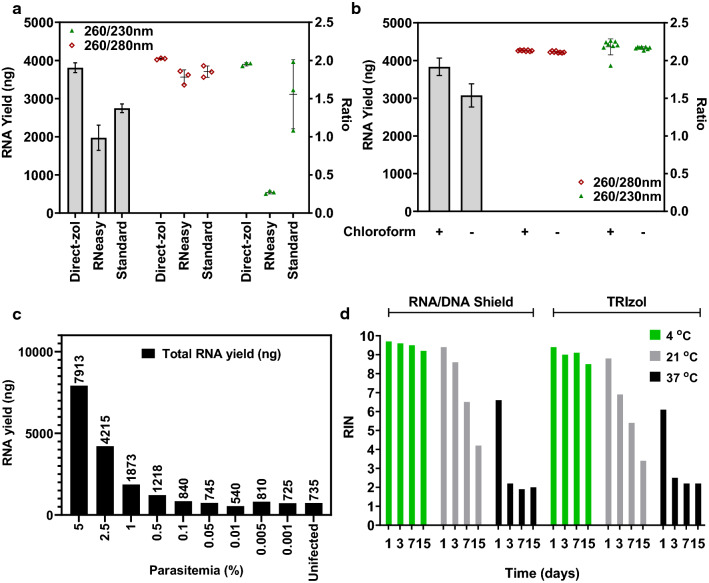
Optimization of RNA extraction and sample preservation methods for *P. falciparum* infected blood samples.** a** Comparison of RNA extraction methods. Total RNA yields, obtained from 500 µl of packed iRBCs (12 hpi, ring stage, 2% parasitaemia) are shown in grey bars. RNA purity assessed with Nanodrop at 230, 260 and 280 nm wavelengths shown as ratios. **b** Effect of chloroform supplementation on total yield and purity of RNA extracted from 500 µl of packed iRBCs (12 hpi, ring stage, 2% parasitaemia) using Direct-zol extraction method. Chloroform presence or absence is indicated below the graph as + or—respectively. RNA purity assessed with Nanodrop at 230, 260 and 280 nm wavelengths shown as ratios. **c** Expected total RNA yields obtained from 500 µl of packed iRBCs infected with a range of parasitaemia (12 hpi, ring stage) obtained by serial dilution of initial parasitaemia of 5%. RNA was extracted from 500 µl of uninfected RBCs as a negative control. **d** Efficacy of TRIzol^TM^ reagent and DNA/RNA Shield^TM^ in preservation of RNA extracted from iRBC (20 hpi, young trophozoites, 8% parasitaemia) stored in either TRIzol^TM^ or DNA/RNA Shield^TM^ were subjected to different temperature conditions for varying periods of time. RNA from samples was extracted using Direct-zol method and analysed on Agilent Bioanalyzer. RIN values were obtained for each condition and are shown here

The main prerequisite for studying genome-wide transcriptional profiles (transcriptomes) is the ability to obtain total RNA extracts that are representative of the exact abundance of all messenger as well as other RNA species in the cell. These RNA isolations must be of sufficient quality to support subsequent transcriptome-wide amplifications (TWA) and readout platforms thus allowing genuine measurements of RNA abundance in a particular cellular/biological system. The vast majority of *P. falciparum* RNA studies conducted in the past two decades utilized a guanidinium thiocyanate-phenol-based reagent, TRIzol™, for cell lysis and subsequent preservation of all nucleic acid molecules (DNA/RNA). Hence, several RNA extraction methods that provide the most optimal procedure for RNA isolation from *P. falciparum* TRIzol™-based cell lysates were assessed. These included the Direct-zol-96 RNA kit (Zymo) further referred to as “Direct-zol”; the RNeasy 96 Universal Tissue Kit (Qiagen) subsequently referred to as “RNeasy” and the previously described isopropanol precipitation method further referred to as “STANDARD” [15]. Starting with 500 µl of iRBCs with 2% ring-stage (12–14  hpi) *P. falciparum* infection, cells were lysed in 10 volumes (i.e. 5 ml) of TRIzol™ and supplemented with 2 volumes (i.e. 1 ml) of chloroform. Aqueous (top) phase was mixed 1:1 (v/v) with 100% ethanol and mixtures were applied on silica columns as described in manufacturer’s manual, or precipitated in ice-cold isopropanol as described previously for STANDARD extraction [15]. Direct-zol kit generated the highest RNA yield of 3.8 µg (SD 0.13, n = 3) followed by the STANDARD isopropanol precipitation and RNeasy generating, 2.74 µg (SD 0.11, n = 3) and 1.97 µg (SD 0.33, n = 3), respectively, as measured by fluorescence-based assay (Fig. 1a).

Spectrophotometric estimation of residual protein impurities in the RNA eluents measured by the signal ratio between 260 and 280 nm wavelength (260/280) was > 1.8 which indicated sufficiently high RNA purity. However, the 260/230 signal ratio, indicating potential organic solvent contamination (residuals from TRIzol™), indicated the optimal performance of the Direct-zol method as the ratio values consistently reached ~ 2. In this regard, the Direct-zol method outperformed the RNeasy and STANDARD extraction techniques (Fig. 1a). Furthermore, Direct-zol-based extraction was also compared in the presence or absence of chloroform by extracting total RNA from 500 µl of iRBCs with 2% ring-stage parasites (12–14 hpi, n = 8). Indeed, the inclusion of chloroform in the Direct-zol method improves the RNA yield by around 20% maintaining the same RNA purity (Fig. 1b). Finally, the Direct-zol was highly efficient in extracting *P. falciparum* RNA from samples with low blood volumes, (useful in high-throughput drug assays, see *Discussion*) yielding 130 ng of total RNA (SD 0.6, n = 8) from as little as 8 µl of iRBCs with rings-stage parasites (12–14 hpi, 4% parasitaemia, without chloroform supplementation). Taken together, these results indicate that the tested Direct-zol is a method of choice for high yield and superior quality RNA extractions from TRIzol™ lysates of *P. falciparum*-infected blood samples (and possibly other *Plasmodium spp.*).

An important factor for RNA extraction from blood samples is the overall yield of *P. falciparum* RNA from varying parasite loads in individual malaria infections. It is necessary to estimate what parasitaemia levels can generate sufficient parasite RNA quantities (purity and intactness) for downstream transcriptomics analysis. To test this, RNA yields were determined from 500 µl of iRBCs infected with *P. falciparum* ring-stage parasites (12–14 hpi), serially diluted from 5 to 0.001% parasitaemia (Fig. 1c). Here, Direct-zol was used as an extraction method and the total RNA yield was quantified using a fluorescence-based assay. The RNA yield from 5% ring stage samples was 7.9 µg (the number approaching maximum binding capacity of the column) with a subsequent drop to 0.84 µg from 0.1% iRBCs. From 0.05% to 0.001% the RNA yield readings leveled between 0.75–0.54 µg, which also reflected the total RNA yield from uninfected RBCs. These results suggest that Direct-zol can purify *P. falciparum* total RNA along with the host RNA residing in the iRBCs and uninfected RBCs present within the samples.

Since the tested Direct-zol extraction method uses TRIzol™ as a tissue homogenizing solution, its capacity for RNA preservation in lysates of iRBCs was assessed. This would eliminate the need for other (preserving) reagents, thus reducing steps in the extraction protocol. To begin with, two reagents: TRIzol™ (Invitrogen), and dedicated commercially available nucleic acid protective solution DNA/RNA Shield™ (ZYMO) further referred to as DNA/RNA Shield™, were compared. It is important to note that DNA/RNA Shield™ is fully compatible with TRIzol™ reagent and Direct-zol method, as stated in manufacturer’s description. In the first step, the effect of temperature and duration for which iRBCs lysates can be stored in these reagents without any significant RNA degradation were investigated. For these experiments, 50 µl of RBCs infected with 8% young trophozoite-stage parasites (20 hpi) was homogenized either with 500 µl of DNA/RNA Shield™ or with 500 µl of TRIzol™.

Samples were incubated for 1, 3, 7, and 15 days at 4, 21, and 37 °C. The RNA was subsequently purified with the Direct-zol method described above without chloroform supplementation. For an assessment of RNA degradation, we have applied the RIN assay from Agilent Bioanalyzer [18]. RIN algorithm estimates RNA quality by measurements of 28S:18S rRNA ratio, as well as several other factors, such as size of 5S rRNA, area under the electropherogram curve, and the ratio of rRNA peak heights to marker peak height [18] Overall, both TRIzol™ and DNA/RNA Shield™ performed similarly well in preserving the RNA at 4 °C for up to 15 days as shown by RIN numbers approaching the maximum value of 10 (Fig. 1d). DNA/RNA Shield™ preserved RNA better at 21 °C for up to 3 days but neither of the reagents could support RNA preservation past that. None of the tested preserving agents could protect *P. falciparum* RNA at 37 °C over 1 day (Fig. 1d and Additional file 2: Fig. S1). Taken together, our results suggest that both TRIzol™ and DNA/RNA Shield™ could protect *P. falciparum* RNA for up to 15 days at 4 °C (e. g. refrigerator) and no more than 3 days at 21 °C, before the lysates must be processed or transferred to -80 °C to store indefinitely. Given the equal performance of the two preserving reagents tested here, we selected TRIzol™ as lysis and preserving agent for subsequent experiments due to its cost-effectiveness, practicality, and application universality.

**Fig. 2:**
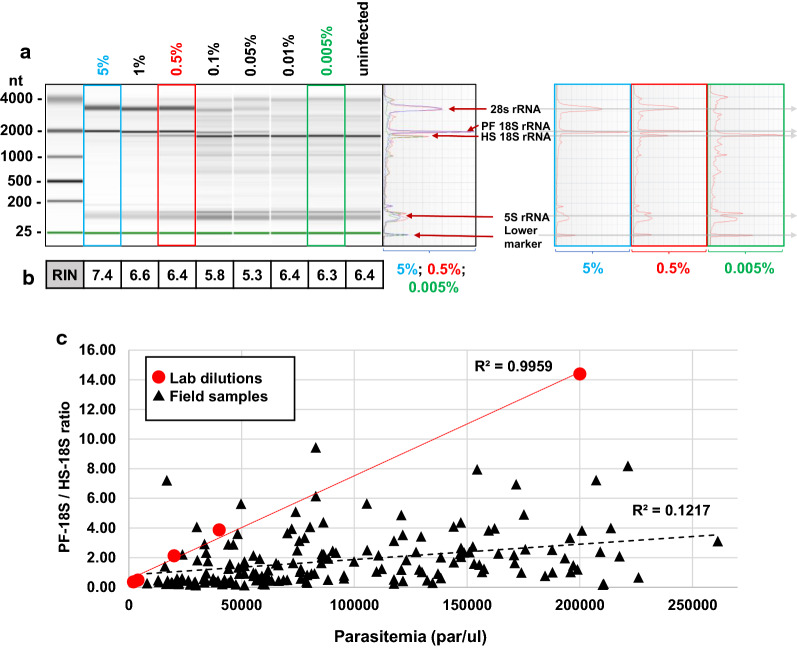
RNA integrity analyses for *P. falciparum* in vitro and field samples using Agilent Bioanalyzer Platform.** a** Agilent Bioanalyzer gel and electropherograms showing comparison of RNA extracted from serially diluted *P. falciparum* iRBCs (12 hpi, ring stage) demonstrating difference in migration speeds of human (HS) and parasite (PF) 18S ribosomal subunits. Range of parasitaemia was obtained by serial dilution of initial parasitaemia of 5%. Uninfected RBCs are shown as a control. Individual and combined electropherograms for 5, 0.5 and 0.005% iRBCs are shown in blue, red and green rectangles in the right side panel. **b** Comparison of Agilent Bioanalyzer RIN values of samples from subfigure (**a**). **c** Correlation between parasitaemia and 18S-Pf/18S-Hs peak height ratio. Peak height values were obtained from Bioanalyzer electropherograms. Lab dilutions (red dots) are 3D7 RNA samples shown in subfigure (**a**); Field samples (black triangles) correspond to 171 RNA samples extracted from malaria patient blood collected during TRAC2 study. Parasitaemia were estimated by Giemsa smear microscopic readings. In order to scale the data, the number of iRBCs per microlitre of whole blood (par/µl) for 3D7 Lab dilutions has been calculated from percentage of iRBCs assuming 40% average haematocrit (e.g. 1% parasitaemia ~ 40,000 par/ µl)

To test whether the derived RNA extraction method performs equally across a wide range of parasitaemia, seven dilutions of the identical 5% iRBCs sample (rings, 12–14 hpi) were prepared to obtain a range of parasitaemia from 5 to 0.005% and uninfected RBCs. Samples were extracted using Direct-zol method and equal amounts of total RNA (100 ng) were analysed on Agilent Bioanalyzer (for details see Additional file 1: Supplementary Protocol). Electropherograms and the RIN values for each sample were obtained. As shown in Fig. 2a, electropherograms of RNA isolated from high parasitaemia samples (i.e. 5 to 1%) contained unique distinct peaks that presumably correspond to the *P. falciparum* 28S, 18S and 5S rRNA transcripts. At lower parasitaemia (i.e. 0.5 to 0.05%), the fluorescence intensity (manifested as peak height on electropherograms) of the parasite-specific rRNA peaks progressively diminish. This is particularly evident for the *P. falciparum* and human 18S rRNA peaks that migrate approximately 1 s apart of each other but are fully distinguishable in the electropherograms (Fig. 2a, right panels). At the lowest parasitaemia tested (e.g. 0.01–0.005%), *P. falciparum* 18S rRNA peak approaches and subsequently “sinks” below the detection limit while the human 18S rRNA transcript dominates the electropherogram profile. As a result, RIN values for the RNA extracts from high and low parasitaemia are higher, than those observed for medium parasitaemia (i.e. 0.1–0.05%), likely due to the apparent dual peaks (Fig. 2b). Taken together, Direct-zol RNA isolation method is applicable to a range of parasitaemia for purifying high-quality RNA originating from both, *P. falciparum* parasites and the host erythrocytes simultaneously.

The presence of two distinctive peaks of the parasite and human 18S rRNA on the Agilent Bioanalyzer electropherogram gives the possibility to use these to estimate the iRBCs concentrations within the RBCs sample. Indeed, the ratio of 18S *Plasmodium* peak height to 18S human peak height (i.e. 18S-Pf/18S-Hs) correlates linearly with parasitaemia for in vitro cultured *P. falciparum* parasites in which the erythrocytes have been purified from other blood components (Pearson Correlation Coefficient (PCC) = 0.99, *p* = 0.11 × 10^–3^, n = 5) (Fig. 2c, red circles). In clinical samples derived from the TRAC2 study, weaker but significant correlations were observed between the 18S-Pf/18S-Hs ratio and parasitaemia (PCC = 0.35, p = 0.29 × 10^–5^, Spearman Correlation Coefficient (SCC) = 0.48, *p* = 0.36 × 10^–10^; n = 171) (Fig. 2c, black triangles). This could be due to parasitaemia not being the unique contributor to 18S-Pf/18S-Hs in the field samples. For example, the presence of host RNA from remaining white blood cells (WBCs) that were not completely removed from the sample could significantly alter the 18S-Pf/18S-Hs peak height ratio. Additionally, older age of the parasites with more RNA present in the cell may cause increased levels (manifested as increased signal intensity) of *P. falciparum* 18S rRNA peaks which can significantly alter 18S-Pf/18S-Hs peak height ratios. Therefore, predicting levels of iRBCs in peripheral blood samples based on the 18S-Pf/18S-Hs ratio gives only limited confidence presumably due to parasite stage distribution and broad variation in WBC depletion that is objectively achievable in varying conditions of field laboratories.

### Whole transcriptome amplification from *P. falciparum* blood-stages total RNA

**Fig. 3:**
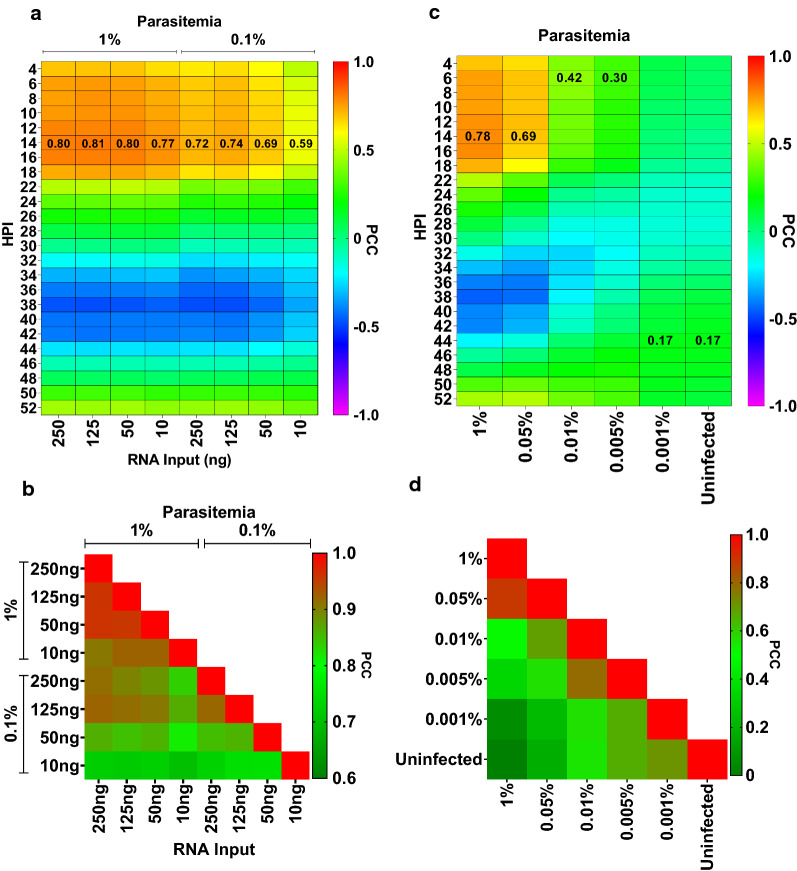
Effect of RNA input and parasitaemia on transcriptome correlations of *P. falciparum *in vitro samples. **a**, **b** Heatmaps depicting correlations between whole transcriptome amplified from varying RNA inputs (for both, low (0.1%) and high (1%) parasitaemia) and **a** 3D7 IDC reference transcriptome, or **b** each other. **c**, **d** Heatmaps depicting correlations between whole transcriptome amplified from varying parasitaemia samples and **c** 3D7 IDC reference transcriptome, or **d** to each other. A constant total RNA input of 250 ng was used across all parasitaemia in (**c**) and (**d**). All data have been obtained using spotted oligo microarray hybridization. PCC values are shown here with maximum correlation coefficients marked as numerals in (**a** and **c**). *HPI =* hours post invasion

Once total RNA has been extracted from iRBCs, gene expression profiling requires synthesis of cDNA through in vitro reverse transcription. Here, a modified version of the Switching Mechanism At the 5′ end of the RNA Transcript (SMART-seq2) method was utilized which allows for amplification of full-length cDNA molecules from single-cell or low RNA inputs of as little as few picograms [19]. First, the modified SMART-seq2 protocol was applied to a range of RNA inputs starting from as low as 10 ng up to 250 ng of total RNA isolated from RBCs infected with 1% or 0.1% identical *P. falciparum* 3D7 cultures (12–14 hpi) (for details see Additional file 1: Supplementary Protocol). To assess the usefulness of the amplified product to study parasite gene expression, microarray hybridization was performed with the amplified cDNA and compared to a high-resolution *P. falciparum* 3D7 strain reference transcriptome generated from 24 time points collected across the IDC (Microarray GEO Acc. No GSE149865; RNA-Seq GEO Acc. No GSE150484). Indeed, transcriptomes generated with various RNA inputs from *P. falciparum* iRBCs with 1% parasitaemia maintained consistently high correlation with the 3D7 IDC reference transcriptome (PCC ~ 0.8) (Fig. 3a) and to each other (Fig. 3b) for as low as 10 ng of total RNA input. This result indicates that when applying Direct-zol extraction combined with SMART-seq2 amplification using *P. falciparum* iRBCs samples with parasitaemia ≥ 1%, as little as 10 ng of total RNA is sufficient to generate reliable transcriptome profiles. On the other hand, when analysing samples with a parasitaemia of 0.1%, a drop in correlations to 3D7 IDC reference transcriptome was observed even for total RNA inputs of 250 ng (PCC ~ 0.7).

Increasing initial RNA input proved to be beneficial for low, but not high, parasitaemia samples (Fig. 3a). RNA input of 50 ng in 0.1% ring stage parasitaemia proved to be a minimum for generating genuine transcriptome profiles (PCC > 0.68) as inputs below 50 ng reduced correlation to reference 3D7 transcriptome (Fig. 3a) and to higher RNA input samples (Fig. 3b) as evidenced in a drop of PCC values. These results indicate that the main factor affecting transcriptome amplification is not the initial RNA input but the percentage of uninfected RBCs and thus human RNA material in the starting RNA sample.

To explore this in greater detail, transcriptomes of serially diluted ring-stage parasites with parasitaemia ranging from 1% to 0.001% (12–14 hpi) were generated using a consistent RNA input of 250 ng. After correlating them to the reference 3D7 IDC transcriptome, a severe reduction in PCC-based correlations and erroneous parasite age estimations were observed for samples below 0.05% parasitaemia (Fig. 3c). This is possibly due to the overwhelming amount of human cDNA present in the total amplicon mix derived from low parasitaemia samples that interferes with both amplification and microarray hybridization. Indeed, in the Agilent Bioanalyzer electropherogram of cDNA amplified from the 0.1% or lower parasitaemia samples (the microarray target material), two sharp cDNA peaks (around 700 bp) were noticed and were determined to represent amplified cDNA from human haemoglobin A (HBA) and haemoglobin B (HBB) transcripts (Additional file 2: Fig. S2a) via capillary sequencing. The HBA and HBB peaks are also observed in the cDNA originating from 1% parasitaemia samples; however, these are accompanied by a broad cDNA peak ranging from 800 bp to 5 kb that represents the amplified *P. falciparum* cDNA products (Additional file 2: Fig. S2b). In highly parasitized samples (e.g. 10%), the entire electropherogram is composed solely of *P. falciparum* cDNA in the broad peak between 800 bp to 5 kb, and no dominant HBA and HBB peaks are seen (Additional file 2: Fig. S2c). Given that 0.1% parasitaemia iRBCs can yield RNA extracts that genuinely reproduce the parasite transcriptome, it suggests that the presence of the human material (e.g. HBA and HBB transcript) does not interfere with microarray hybridization if enough parasite cDNA is available.

At the sequencing step, analysis of exonic x-coverage and proportions of parasite unique reads to human unique reads across the two dilutions of iRBCs was performed. It was estimated that for ring-stage parasites (12–14 hpi) and high parasitaemia (1%), 24 samples can be safely multiplexed per lane of Illumina HiSeq4000 in order to obtain 5 × 10^6 ^unique *P. falciparum* reads (approx. 1.5 GB) per sample (Additional file 2: Fig. S3). This generated high correlations to the reference transcriptome (PCC ~ 0.8) during comparative transcriptomics analyses (Additional file 2: Fig. S4). When multiplexing 24 samples with 1% parasitaemia, close to 200 × exonic coverage on average (Additional file 2: Fig. S5) was obtained. Decreasing parasitaemia to 0.1% resulted in non-linear drop of parasite-specific reads and average exonic x-coverage (~ 75%). That, in turn, caused lower correlation values to the reference transcriptome (PCC ~ 0.6) (Additional file 2: Fig. S4). Taking all these data into account and based on experience with RNA-seq data derived from field samples (unpublished data), internal standards were created when performing comparative transcriptomics where a safe margin of 3 × 10^6^ parasite unique paired-end reads and 100 × average x-coverage are advised in order to generate reliable conclusions.

Lastly, not much sequencing reads mapped to intergenic regions suggesting negligible genomic DNA (gDNA) presence (Additional file 2: Fig. S5)**.** However for assays/studies very sensitive to gDNA contamination, or if leucocyte depletion was not performed initially, a fast and simple additional DNase treatment step has been included in the pipeline (See Additional file 1: Supplementary Protocol).

### High-throughput extraction of total RNA using magnetic beads

The next step in this endeavour was to derive a high throughput method that could facilitate RNA isolation on a large scale using robotic platforms. This was particularly applicable to the population transcriptomic effort during the TRAC2 project when RNA was extracted from more than 1000 peripheral blood samples. Hence, the utility of RNA extraction using TRIzol™-compatible magnetic bead-based purification using Direct-zol-96 MagBead RNA (ZYMO) further referred as “Direct-zol MagBeads” was explored. RNA extraction was performed on 50 µl of iRBCs (4% parasitaemia, 6 hpi, in 300 µl of TRIzol™) using Direct-zol MagBeads method (n = 3), or, Direct-zol method (n = 3) described above, with or without chloroform supplementation. Compared to the column-based purification method, the magnetic bead purification yielded 14–19% lower amounts of RNA from the same starting material (Additional file 2: Fig. S6). Adding chloroform to the homogenate slightly improved yields in both methods. RNA purity and RINs analysis confirmed a comparable quality of the resulting RNA isolations with 260/280 and 260/230 ratios in acceptable range (Additional file 2: Fig. S6) and RIN_Direct-zol_ = 7.7 (SD 0.6) and RIN_Direct-zol MagBeads_ = 7.5 (SD 0.8). Finally, the bead-isolated RNA supports transcriptomic analysis (microarray) that is comparable to the column based approach with PCC ~ 0.77 to the 3D7 IDC reference dataset (Additional file 2: Fig. S7). These results suggest that the application of the magnetic bead purification approach is a viable option for isolation of *P. falciparum* RNA.

### Assessing the efficiency of described RNA processing pipeline for studying malaria clinical isolates

**Fig. 4:**
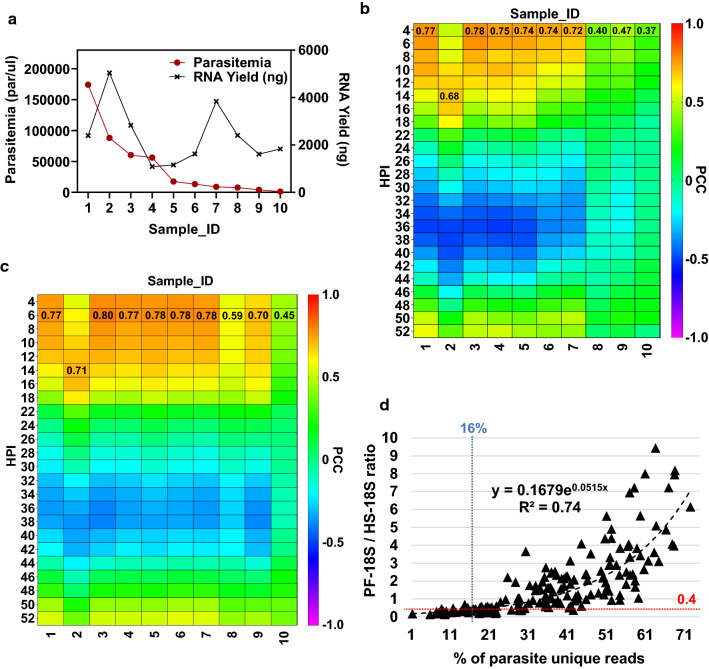
Validation of RNA processing pipeline on malaria-infected clinical samples from TRAC2 study. **a** Giemsa smear estimated parasitaemia and total RNA yields obtained from 200 µl of iRBCs from ten patients' blood samples. **b, c** A heatmap showing correlation of whole transcriptome amplified from patients' blood samples to 3D7 IDC reference transcriptome based on **b** FPKM values obtained from RNA sequencing, **c** spotted oligo microarray hybridization. The highest PCC values are shown in numerals. The same total RNA input of 250 ng was used for reverse transcription of all samples. All ten patients' samples in panel **a**, **b**, and **c** have been selected from various clinical sites across South East Asia and sorted from the highest to the lowest parasitaemia. **d** A scatterplot depicting positive correlation between 18S-Pf/18S-Hs peak heights ratio and percentage of *P. falciparum* unique reads. All data were obtained from Bioanalyzer electropherograms and RNA sequencing of 171 TRAC2 clinical blood samples. Blue and red dotted lines indicate minimum values of the parasite reads proportion (16%) and peak ratio (0.4), respectively, in order to obtain 3 million unique parasite reads when multiplexing 20 samples on Illumina HiSeq 4000 lane.

Development of robust, high-throughput transcriptome analyses employing microarray and/or RNA-seq opens up new opportunities to study molecular mechanisms underlying multiple parasite phenotypes such as drug resistance, fitness associated with diverse drug selection pressures, virulence and transmissibility. This section demonstrates that the proposed RNA processing and amplification pipeline works equally well for malaria clinical samples. First, RNA was extracted from ten selected clinical isolates obtained from the TRAC2 study (iRBCs homogenized in TRIzol™) which gave a wide range of total RNA yields that were not completely proportional to the parasitaemia of the samples (Fig. 4a). This is due to a variable presence of human RNA (e.g. sample no. 7 and 8) or higher median age of the parasites in the peripheral circulation of the patient during blood collection (e.g. sample no. 2). Both of these factors affect the overall RNA yield as measured by fluorescence-based assay. Regardless, for all ten samples, the RIN values fell between 5.9 and 8.3 (median 7.6) (Additional file 2: Fig. S8 and Fig. S9). Sample number 1 to 5 show *P. falciparum* unique reads ranging between 30 to 97% *versus* unique human reads (Additional file 2: Fig. S10). The lower parasitaemia samples 6 to 10, showed less than 15% reads mapping to *P. falciparum* genome uniquely (Additional file 2: Fig. S10 and Fig. S11). Once again, the proportion of parasite reads is not tightly correlated to parasitaemia of the samples possibly due to the presence of excessive human RNA or older parasites (Additional file 2: Fig. S12). For example, sample number 3 with parasitaemia over 60,000 parasites/µl has low unique parasites reads proportion and very visible haemoglobin (Hb) transcripts bands seen on the gel indicating the significant presence of human material (Additional file 2: Fig. S9, bottom panel, orange box). Additionally, no clear parasite-specific 18S rRNA peak is seen on the gel (Additional file 2: Fig. S9, top panel, red arrows). All that would support the possibility of excessive human nucleic acids presence in the sample.

For seven of these samples (No. 1–7) the resulting transcriptome obtained from RNA-seq platform correlated well with the respective time points in the 3D7 IDC transcriptome reference dataset with PCC > 0.7 to early ring stage (except sample 2 that appears to be older) (Fig. 4b). The three remaining samples (No. 8–10) showed low correlation to the reference dataset which sets a practical threshold of parasitaemia for the RNA-seq based transcriptomics in field studies (for the particular sequencing conditions used in this experiment) at approximately 8000 parasites/µl of whole blood when successful CF11 column WBCs depletion was performed. A higher threshold of minimum parasitaemia for clinical isolates as compared to 3D7 in vitro culture may be due to incomplete WBCs depletion and younger parasite age in patient peripheral blood samples. Interestingly, the same samples processed using DNA microarray platform, yielded stronger correlations to the reference 3D7 IDC transcriptome including those with low parasitaemia (samples 8–10) (Fig. 4c). This indicates that DNA microarrays are less sensitive to the presence of contaminating host RNA and supports genuine transcriptomic measurements in clinical samples with as little as 4176 parasites/µl of whole blood (Sample no. 9), only slightly higher than our lab culture-derived microarray-based threshold of 0.05% (approx. 2000–3000 parasites/µl).

Furthermore, after sequencing 171 clinical samples from the TRAC2 study (unpublished data), it was found that the 18S-Pf/18S-Hs ribosomal peak-height ratio can be used to estimate the proportion of unique *Plasmodium* reads to total reads with good accuracy before sequencing experiments (Fig. 4d). Notably, a strong exponential correlation of 18S-Pf/18S-Hs peak ratio to the proportion of parasite unique reads (measured as the percent of total reads) (PCC = 0.75, SCC = 0.85, n = 171) was obtained. This provides a key parameter for estimation of the expected proportion of unique parasite reads in the overall RNA-seq dataset. Correlations of unique parasite read proportions to parasitaemia (by microscopy) were much less pronounced (PCC = 0.4, SCC = 0.42), which further highlights the confounding factors for RNA analysis including the presence of human host material and IDC stage representations (see above) (Additional file 2: Fig. S12). Based on the analysis of data obtained from these 171 TRAC2 samples, it can be concluded that in order to obtain 3 million *Plasmodium* unique reads (a safe threshold established for reliable analysis of differential expression of the whole transcriptome), approximately 16% of the total reads must uniquely map to *Plasmodium* genome (for sequencing conditions used in this experiment) (Fig. 4d, blue dotted line). In other words, when the 18S-Pf/18S-Hs ribosomal peak-height ratio value reaches approximately 0.4 (Fig. 4d, red dotted line), it is estimated that at least 16% of total reads will map uniquely to *Plasmodium*. This peak ratio indicator should be applied when estimating how many samples per lane can be multiplexed in order to obtain enough parasite transcriptome coverage.

### CRISPR-Cas9 assisted depletion of haemoglobin transcripts

**Fig. 5:**
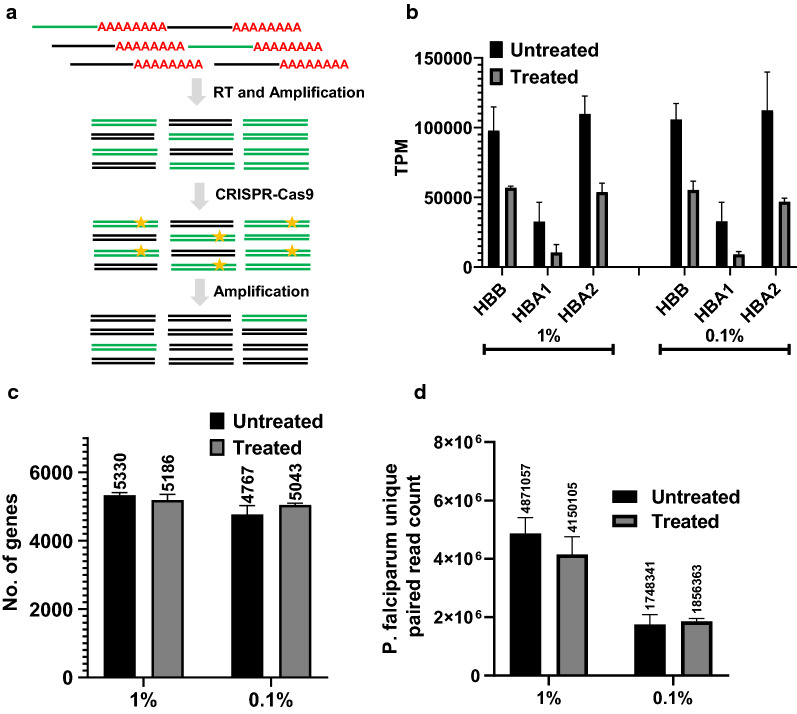
CRIPSR-Cas9 assisted depletion of haemoglobin transcripts. **a** A schematic diagram of in vitro CRISPR-Cas9 assisted depletion of haemoglobin transcripts. Green and black coloured poly-A tail mRNA represent haemoglobin transcripts and parasite transcripts respectively. After reverse transcription and amplification (5 cycles of PCR), gRNA-Cas9 assisted depletion of haemoglobin cDNA was performed (yellow stars). **b** Successful depletion of HBB, HBA1, HBA2 haemoglobin transcripts shown as transcripts per million (TPM) as measured by RNA sequencing for 1 and 0.1% ring stage iRBCs. **c** Total number of *P. falciparum* transcripts detected in 1 and 0.1% ring stage iRBCs in the presence or absence of gRNA-Cas9 assisted depletion. **d** *P. falciparum* unique reads count expressed as TPM shown in the presence or absence of gRNA-Cas9 assisted depletion. For all graphs, untreated samples (black) were subjected to 19 cycles of amplification only, treated samples (grey) were amplified for 5 cycles, treated with gRNA-Cas9 and then amplified for additional 14 cycles

Detailed inspection of our RNA-seq data revealed that the majority of the human mapped reads belong to the Hb gene family (majorly HBA1, HBA2 and HBB). Hence, it is feasible to speculate that suppression of the HBA1, HBA2, and HBB transcript amplification during TWA can significantly improve the overall coverage of the *P. falciparum* transcriptome, particularly in samples with a high content of host material. To do so, cDNA libraries were treated with sequence-specific guide RNA (gRNA) and Cas9 nuclease to perform in vitro selective depletion of targeted Hb sequences. This method has been originally reported as Depletion of Abundant Sequences by Hybridization (DASH) but here, the original DASH protocol was modified to suit the presented pipeline [20]. Specifically, in order to deplete Hb transcripts, pre-amplification PCR was performed in two steps. The first-strand cDNA was amplified for 5 cycles using KAPA HiFi HotStart ready mix. Subsequently, the purified amplicon was treated with gRNA-Cas9 complex (1:1000 ratio of cDNA:gRNA) followed by another 14 cycles of PCR (Fig. 5a). This modification allowed us to start with higher cDNA amount for gRNA-Cas9 treatment thus improving cDNA recovery after bead-based purification of the depleted amplicon.

To assess the performance of CRISPR-Cas9 assisted Hb transcript depletion, both high (1% rings, 12-14hpi, n = 3) and low (0.1% rings, 12-14hpi, n = 3) parasitaemia samples derived from identical parasite population were treated and the corresponding transcriptomes were analysed by RNA-seq. A significant reduction in the read count (shown as transcripts per million, TPM) for HBA1, HBA2 and HBB transcripts was observed in CRISPR-Cas9 treated 1% and 0.1% parasitaemia cDNA samples when compared to untreated amplified cDNA (Fig. 5b). The data revealed more than 70% depletion of Hb transcripts after gRNA-Cas9 treatment. The total number of parasite transcripts detected and parasite unique read count improved for the lower (0.1%) parasitaemia samples (Fig. 5c, d) with 276 new *P. falciparum* transcripts being detected after Hb transcript depletion. These new transcripts belong to the low-abundance group with averaged median TPM of 1.36 (SD 0.75) (Additional file 2: Fig. S13). The “missing transcripts” in treated 1% parasitaemia samples also belong to the low-abundance group with averaged median TPM of 1.8 (SD 0.91) (Additional file 2: Fig. S13). Taken together, the above observations suggest that CRISPR-Cas9 depletion of unwanted transcripts would be most applicable to low parasitaemia samples.

## Discussion and practical considerations

With the rise of the sequencing technology, whole transcriptome analyses became accessible to most laboratories worldwide. However, in order to avoid the artefacts and obtain genuine transcriptional variation between sampling sites, it is crucial to work only with the highest quality RNA. Amongst many challenges facing researchers working with malaria blood samples are, for instance, high ambient temperature and long transportation time from the field sites to the laboratory [21]. All these can have a detrimental effect on RNA integrity. As different mRNA transcripts may degrade at different rates it can result in drawing of false conclusions from final data [22]. Here, a step-by-step guide on how to obtain high-quality RNA from both laboratory and field malaria-infected blood samples is presented that is fully compatible with transcriptomic analyses. Since modern field studies of malaria epidemiology and physiology can involve a large number (> 1000) of subjects [23], this protocol has been designed so that it can be streamlined for high-throughput projects and robotic handling can be applied.

For RNA isolation, the developed pipeline focused on several major issues that every transcriptomic study may face. Obtaining sufficient amounts of RNA free of contaminants is of utmost importance in any transcriptomic study. Based on extensive prior experience there are numerous challenges with *P. falciparum* or other *Plasmodium spp.* RNA isolations. First, the high viscosity of the initial *Plasmodium* iRBCs lysates can lead to suboptimal operation of silica-based columns (used in the vast majority of RNA purification products), thus affecting the overall RNA yield and integrity. Second, the final RNA eluent can contain a high amount of impurities such as haem, lipids, and proteins that could inhibit subsequent RNA processing (such as amplification) [24]. Third, the final RNA extract can/will contain some fraction of human host RNA originating from remaining leukocytes (in clinical samples) and host erythrocytes (both clinical and laboratory samples). These problems stem from the nature of the material from which intraerythrocytic stage malaria parasites RNA is extracted (iRBCs) that is a blood fraction depleted of plasma and (most of) WBCs.

There are approximately 4–6 million RBCs in a microlitre of human blood and approximately twice as much in “packed RBCs” suspensions that are approaching 100% haematocrit. This large number of cells and its unusually high protein (mostly haemoglobin) content is likely the main reason why, upon lysis, blood samples become highly viscous, rich in protein, lipid and free haem, all of which can impede the subsequent RNA extraction methods (such as silica-based columns). To avoid this in the past, most of the methods for parasite RNA extraction relied on TRIzol™/chloroform homogenization followed by cold isopropanol or ethanol precipitation [25]. This procedure generates high quality and integrity RNA, efficiently removing proteins, lipids and DNA from the final eluent. However, this “manual” approach is subjected to a large variability of yields, purity and integrity and cannot be easily streamlined to studies involving large number of samples. Moreover, it requires a certain amount of starting cell material for efficient separation of the phases and as such is unsuitable for very low blood volume samples (e.g. 8 µl). Here, the authors aimed to overcome such variabilities by combining standard TRIzol™ extraction with silica-based column or magnetic beads RNA purification that would ensure high reproducibility and thus a possibility of standardization. For that, two extraction methods were adapted using silica-based columns (Direct-zol, RNeasy), that were deemed TRIzol™ compatible by their manufacturers. As shown by the data, both of them gave consistently reproducible results that were comparable to, or better than standard isopropanol precipitation used until now. Both Direct-zol and RNeasy produced RNA of sufficient yield with the former slightly outperforming the latter in RNA purity. In addition, the Direct-zol method supports RNA elution in volumes as low as 10 µl, which makes it more practical, especially when working with limited blood volumes. Moreover, as stated by the manufacturer and confirmed by our electropherogram traces, Direct-zol was able to extract even small RNA fragments (> 17nt) that allowed to capture small RNA species and 5S ribosomal RNA subunits. In addition to single-column format for low-throughput studies, Direct-zol can be also applied in a 96-well format, thus making it suitable for large, automation-aided studies. In order to make the extraction process even faster and more accessible beads-based, TRIzol™ compatible method that does not require expensive centrifuges or liquid handlers were also adapted. Overall, the results indicate that beads isolate RNA of similar quality compared to the Direct-zol column-based method with only minor loss in yield.

Most of optimization analyses in this study were conducted with 500 µl of iRBCs which represents a standard large volume sample that is near the capacity of a single column purification. However, it has been shown here that the same purification protocol can accommodate as little as 8 µl of iRBCs to generate sufficient RNA material necessary for a transcriptomic assay. Based on that, it is deduced that this RNA purification pipeline could support analyses of parasite transcriptome in small in vitro cultures, such as those performed in 96-well plates. This could allow conducting large studies involving, for instance, parasite transcriptome response to broad arrays of drugs at variable concentrations. Similarly, studies of in vivo transcriptomes may be conducted with blood volumes as low as 20 µl or possibly less (given the standard 40–45% patient’s haematocrit). Hence, this protocol may support transcriptomic analyses from standard finger-prick rather than traditional venous blood sample collection, given that robust leukocyte depletion can be achieved. Overall, the entire pipeline could support a variety of other applications, such as transcriptomics of mouse malaria blood stages, host-parasite interaction analyses, and, ultrasensitive diagnostics of *Plasmodium* infections in the field, where either only very limited starting material is available or where assay sensitivity and/or high-throughput processing are of much importance [26–30].

As mentioned, leukocyte depletion is the main consideration for obtaining high quality *Plasmodium* RNA suitable for transcriptomic analysis [31]. It is crucial to preserve infected erythrocyte lysate and avoid any degradation during the isolation step but most importantly also to minimize the host RNA interference during amplification and transcriptome detection by either DNA microarrays or RNA-seq. The results of this study show that standard CF11-based leukocyte depletion (or equivalent) from clinical samples provide RNA that supports *P. falciparum* reliable transcriptome analyses using both microarray and RNA-Seq platform, for samples with parasitaemia of 8000 parasites/µl of whole blood or more. However, even after successful depletion of leukocytes, the sequencing data indicates that there is still an excess of Hb transcripts from RBCs which saturates the sequencing platform. Thus, a modified DASH method for the depletion of human transcripts is presented here. Although as a proof of the principle this study focused on depletion of the most abundant transcripts present in malaria transcriptome analyses (HBA and HBB), this protocol could be applied to any unwanted transcripts using a relevant gRNA. Based on the data, the use of CRISPR mediated depletion is recommended mostly for low parasitaemia samples.

As *Plasmodium* transcriptomic studies become more important both in the field and in the lab, larger sample numbers are being generated [32, 33]. In order to increase the throughput and decrease variability (inherent to manual handling) between samples, sample processing automation should be gradually introduced in every laboratory that handles a large number of specimens. Although there is a growing number of studies incorporating liquid handlers for RNA extraction and processing, using automation for whole transcriptome analysis is still generally rare, and has not been reported so far in malaria field [34–37]. Considering all of the above, the presented protocol is designed to fully fit the automated approach from start to end. For the purpose of this study, the Direct-zol method was successfully adapted to Tecan robotic platform with integrated centrifuge and created a fully walk-away script taking approximately 2 h to complete the extraction of 96 samples.

Since automation-compatible, or even standard centrifuges, necessary for column-based extractions, can be very expensive and not available in all locations, a bead-based, TRIzol™-compatible RNA extraction approach was also tested. Similarly, purification of amplified cDNA can also be performed using DNA purification magnetic beads. Typically, column-based purification has a low-throughput whereas bead-based purification is easily compatible with medium and high-throughput sample processing when used with 96-well low elution volume magnetic plate. The DNA beads purification protocols was successfully adapted to Hamilton liquid handling platform for a fully walk-away protocol. However, it must be noted, that magnetic beads are impractical and costly when working with very high volume blood samples and have higher elution volumes than columns.

## Limitations

This protocol has been used so far to successfully generate transcriptomic data from three *Plasmodium* species, namely, *P. falciparum*, *Plasmodium cynomolgi*, *Plasmodium vivax*. Presented protocol was tested on, and is most suited for *P. falciparum* studies in both, lab, as well as clinical settings. When studying other species, limitations such as higher GC content (e.g. *P. vivax*) or asynchronous clinical infections (e.g*. Plasmodium knowlesi*) need to be considered. Some of these limitations can be overcome by slight alterations to the existing protocol such as, changing salt concentrations and elongation temperatures during amplification steps to counter higher GC content. Insufficient transcriptome coverage due to low parasitaemia, found very often in clinical samples, can be improved by increasing sequencing depth. Furthermore, various globin/overrepresented transcripts depletion methods can be easily introduced to the protocol in order to increase *Plasmodium*-specific RNA ratio (for example, in vitro CRISPR globin depletion or commercially available rRNA/globin depletion, and library prep kits such as, Zymo-Seq RiboFree™ Total RNA Library Kit (ZYMO) or Ribo-zero Gold Kit (Illumina)). Finally, the study of asynchronous cultures can be performed by enriching for developmental stages of interest using FACS-based cell sorting technology. The presented amplification protocol was based on ultra-sensitive single cell amplification method and can be easily integrated into sorting protocols.

## Conclusions

In conclusion, a robust RNA processing and transcriptome analysis pipeline applicable to both laboratory strains and field samples has been developed  that can be easily streamlined for robotic handling. The presented protocol allows for the usage of commonly used RNA preserving reagents and is compatible to work with a wide range of parasitaemia and starting RNA material. Furthermore, the flexibility to adapt this protocol to silica-column or magnetic beads technology demonstrates its usefulness to a wide variety of laboratory settings/infrastructure. To facilitate an easy application of this protocol, a detailed step-by-step protocol from RNA extraction to transcriptomic data processing has been presented. Although this pipeline has only been tested for *Plasmodium*, this protocol could possibly be applied to study other eukaryotic microorganisms present in the bloodstream.

## Supplementary information

**Additional file 1. **Supplementary Protocol.

**Additional file 2. **Supplementary Figures.

## Data Availability

**Datasets accession numbers** The datasets generated and analysed during the current study are available in the GEO repository: 3D7 P. falciparum Life Cycle Microarray data—GSE149865; 3D7 P. falciparum Life Cycle RNA-seq data—GSE150484; Microarray data from Field Samples and serial dilutions of 3D7 strain: GSE151754. RNA-Seq data from Field Samples and Crispr-Cas9 Depletion of globins in 3D7 strain—GSE151557. **Scripts** All scripts used for microarray normalization and probe averaging are available from authors upon request.
