# Supplementary material for: A comprehensive RNA handling and transcriptomics guide for high-throughput processing of Plasmodium blood-stage samples
Source: Malar J. 2020 Oct 9;19:363. doi: 10.1186/s12936-020-03436-w (PMC7547485; doi:10.1186/s12936-020-03436-w)
Supplement: Supplementary file 1 — Additional file 1. Supplementary Protocol. [file 12936_2020_3436_MOESM1_ESM.docx]

**SUPPLEMENTARY PROTOCOL**

**A comprehensive RNA handling and transcriptomics guide for high-throughput processing of Plasmodium blood-stage samples.**

Michal Kucharski^1†^, Jaishree Tripathi^1†^, Sourav Nayak^1^, Zhu Lei^1^, Grennady Wirjanata^1^, Rob W. van der Pluijm^2,3^, Mehul Dhorda^2,3,4^, Arjen Dondorp^2,3^, Zbynek Bozdech^1*^

^1^ School of Biological Sciences, Nanyang Technological University, Singapore, 637551

^2^Mahidol Oxford Tropical Medicine Research Unit, Faculty of Tropical Medicine, Mahidol University, Bangkok, Thailand.

^3^Centre for Tropical Medicine and Global Health, Nuffield Department of Medicine, University of Oxford, Oxford, United Kingdom.

^4^WorldWide Antimalarial Resistance Network – Asia Regional Centre, Bangkok, Thailand.

^†^Authors contributed equally to this work

^*^Corresponding author: [zbozdech@ntu.edu.sg](mailto:zbozdech@ntu.edu.sg)

**Abbreviations**

RT room temperature (21^o^C)

WBCs white blood cells

RBCs red blood cells

iRBCs infected red blood cells

PCR polymerase chain reaction

s seconds

min minutes

RIN RNA Integrity number

gDNA genomic DNA

cDNA complementary DNA

aa-dUTPs 5-aminoallyl-dUTP

aa-dNTPs aminoallyl-dNTPs

gRNA guide RNA

EB elution buffer

HBA Hemoglobin A

HBB Hemoglobin B

dsDNA double stranded DNA

qPCR quantitative polymerase chain reaction

IDC Intraerythrocytic developmental cycle

**MATERIALS**

**Reagents**

| **Reagent name** | **Manufacturer** | **Catalogue number** |
| --- | --- | --- |
| Ethanol 99.5-99.8 %, absolute | Merck | 1.00983.2500DE |
| RNaseZap™ | Ambion | AM9780 |
| TRIzol™ Reagent | Invitrogen | 15-596-018 |
| Nuclease-free water | Ambion | AM9932 |
| HL-dsDNase | ArticZymes | 70800-202 |
| 50mM MgCl2 solution | Bioline | BIO-37026 |
| dNTP set (100 mM each) | Thermo Scientific | R0181 |
| SuperScript™ II reverse transcriptase 200 U/µl | Invitrogen | 18064014 |
| 5X First-Strand Buffer | Invitrogen | 18064014 |
| DTT 0.1 M | Invitrogen | 18064014 |
| RNaseOUT™ 40 U/µl | Invitrogen | 10777019 |
| Betaine 5 M | Sigma-Aldrich | B0300-5VL |
| MgCl2 1 M | Sigma-Aldrich | M1028-100ML |
| KAPA HiFi HotStart ReadyMix (2x) | Roche | KK2601 |
| AMPure XP beads | Beckman Coulter | A63882 |
| 10 mM Tris-Cl pH 8.5 (EB Buffer) | Qiagen | 19086 |
| *Taq* DNA polymerase 5U/µl | New England Bio labs | M0273L |
| Agilent RNA 6000 Nano Kit | Agilent Technologies | 5067-1511 |
| Agilent DNA 12000 Kit | Agilent Technologies | 5067-1504 |
| Agilent High Sensitivity DNA Kit | Agilent Technologies | 5067-4627 |
| Qubit™ dsDNA Broad Range (BR) Assay Kit | Invitrogen | Q32850 |
| Qubit™ dsDNA High-Sensitivity (HS) Assay Kit | Invitrogen | Q32851 |
| Qubit™ RNA Broad Range (BR) Assay Kit | Invitrogen | Q10210 |
| Nextera® XT DNA Library Preparation Kit (96) | Illumina | FC-131-1096 |
| Nextera® XT Index Kit (24 indexes, 96 samples) | Illumina | FC-131-1001 |
| Cas9 enzymes (20 µM) | New England Biolabs | M0386T |
| Proteinase K (20 mg/ml) | Thermofisher | EO0491 |
| HBA and HBB gRNA (100 µM) | Life Technologies | Custom Ordered |
| Phosphate-Buffered Saline (10x) | 1^st^ Base | BUF-2041 |
| Direct-zol™ RNA MicroPrep (low throughput) | ZYMO | R2062 |
| Direct-zol-96™ RNA (high throughput) | ZYMO | R2056 |
| Chloroform, >99.8% | Fisher Scientific | AC383770025 |
| Plasmodipur Filters | EuroProxima | 8011Filter25u |
| 5-Aminoallyl-dUTP, lyophilized powder | Biotium | 40020-1 |

**Labware**

| **Labware name** | **Manufacturer** | **Catalogue number** |
| --- | --- | --- |
| Eppendorf twin.tec® PCR plate 96 LoBind | Eppendorf | 0030129512 |
| MicroAmp™ Optical Adhesive Film | Thermo Fisher | 4311971 |
| MicroAmp™ Adhesive Film Applicator | Thermo Fisher | 4333183 |
| Cryogenic Microplate seal, Foil, 35 μm, Aluminum | Axygen | AXYGPCR-AS-200 |
| Microcentrifuge tubes, 1.5 ml | Axygen | MCT-150-C |
| Vertex NoStick® Pipette Tips: 10 XL, 20, 100, 200, 1250 μl | SSIBio | 4137NSF, 4237NAF, 4237NBF, 4237NSF, 4347NSF |
| Qubit assay tubes, 0.6 ml | Axygen | AXY-MCT-060-C |
| 15 ml polypropylene conical tubes (falcons) | Greiner | 188271 |
| 96 Well Polystyrene Microplate (300 µl), round bottom clear | Corning | CLS3795-100EA |
| Cryogenic tubes, 1.8 ml, Polypropylene, round bottom, external thread | USA Scientific | 1418-8310 |
| **Labware used for automation** |  |  |
| Conductive filtered tips: 50, 300 μl | Hamilton | 235948, 235903 |
| Reagent tubs with lids | Hamilton | 56694-01 |
| Conductive LiHa filtered tips 1000 μl | Tecan | 10612513 |
| MCA96 filtered tips 200 μl | Tecan | 30038618 |
| Automation Reservoirs | Thermo Scientific | 1064-15-6 |
| 96-well Clear V-Bottom 2 mL Polypropylene Deep Well Plate | Corning | 3960 |

**Equipment**

| **Equipment name** | **Manufacturer** | **Model** |
| --- | --- | --- |
| Mini vortexer | Kenis | Vortex-Genie 2 |
| Thermal cycler | Eppendorf | **Master cycler Pro** |
| Magnetic stand 96 | Alpaqua | Magnum FLX |
| Illumina DNA sequencing instrument | Illumina | HiSeq4000, HiSeq2500, |
| Fluorometer | Thermo Scientific | Qubit 2.0 |
| Automated electrophoresis system | Agilent | Bioanalyzer 2100 |
| Benchtop centrifuge (capable of spinning 96-well plates) | Eppendorf | 5810R  5427 |
| PCR Cabinet | Esco | PCR-4A1 |
| UV-Vis Spectrophotometer | Thermo Scientific | Nanodrop 8000 |
| **Equipment used for automation** |  |  |
| Centrifuge for automation | Hettich | ROTANTA 460 ROBOTIC |
| Robotic platform for extraction | Tecan | Evo 200 |
| Robotic platform for beads purification | Hamilton | Nimbus4 |

**Primers**

All primers were manufactured by Integrated DNA Technologies, Inc. and delivered following manufacturer recommendations. Primers were resuspended in RNase/DNase-free PCR-grade water to 100 µM and then diluted to final working concentrations.

| **Name** | **Sequence** | **Stock concentration** | **Description** |
| --- | --- | --- | --- |
| Oligo-dT_30_VN | AAGCAGTGGTATCAACGCAGAGTACT_30_VN* | 100 µM | Anneals to poly-A tail of mRNA molecule |
| LNA-TSO | AAGCAGTGGTATCAACGCAGAGTACATrGrG+G** | 100 µM | Template switching oligo with locked nucleic acid |
| ISPCR Oligo | AAGCAGTGGTATCAACGCAGAGT | 100 µM | Anneals to universal ISPCR sequence from LNA-TSO and Oligo-dT_30_VN during PCR amplification step after reverse transcription |

***** V = A,C,G; N = A,C,G,C

** rG = riboguanosine; +G = LNA-modified guanosine

**gRNA sequences**

| **Type** | **Gene target** | **Sequence** | **bp** | **Tm** | **Product size** |
| --- | --- | --- | --- | --- | --- |
| Guide RNA | HBA1, HBA2 | 5’-GCUCCUAAGCCACUGCCUGC-3’ | 20 | - | - |
| Guide RNA | HBB | 5’-AGCGAGCUUAGUGAUACUUG-3’ | 20 | - | - |

**PROCEDURES**

**SECTION 1 – HOMOGENIZATION**

- TIMING: depending on the number of samples
- TEMPERATURE: RT / on ice if sample number > 10

**Workspace setup**

- Clean all equipment, fume hood, workstations and bench areas with RNase eliminating reagents (e.g. RNaseZap).
- Use only nuclease-free PCR-grade water, labware and reagents
- Change gloves frequently during the protocol to avoid contamination of samples with RNases.
- When working with large number of samples (>10) it is recommended to keep tubes on ice before and after homogenization to reduce RNase activity.

**Sample Preparation**

- As little as 8 μl of highly infected RBCs can be used. Very young ring stage parasites possess little RNA, hence when working with lab cultures it is important to keep parasitemia of samples high (minimum 2%). This will ensure optimal detection of parasite transcripts and a higher parasite to human read count proportion obtained from the sequencing. To obtain enough RNA material for several experiments and potential repeats preferably 100 μl of iRBCs should be used if possible.
- When working with cell cultures, spin them in swing rotor bucket for 3 min at 2500g, brake 2, RT. Aspirate supernatant and wash iRBCs with 1x PBS (RT) once and spin again.
- When working with whole blood derived from clinical samples, deplete WBCs by centrifugation and aspiration of the buffy coat followed by filtration through CF11 (cellulose powder columns), non-woven polyester filters, or Plasmodipur filters. Please refer to manufacturer’s instructions regarding the protocol.

**Protocol**

- **SAFETY TIP:** Phenol is a volatile and toxic irritant. Perform all steps under fume hood and using personal protective equipment (gloves, mask, safety glasses, and lab coat).
- **CRITICAL:** Working with frozen material.

Although it is possible to work with previously frozen RBCs, we recommend adding TRIzol to fresh blood as it decreases vortexing time and reduces clumping. Vortex or shake samples well. No clumps should be present. Thaw samples fast in 60^o^C water bath (1-2 minutes or until no ice present). Alternatively, samples can be defrosted by leaving them on ice. After thawing samples keep them on ice. Once thawed, samples should not be frozen again.

1. Transfer desired amount of iRBCs to fresh cryogenic tube (for long term storage), 1.5 ml microcentrifuge tube or 96-well plate depending on volume used and if there will be a need for future cryopreservation of homogenized samples.

- *Tip:*

*For liquid nitrogen and deep freezing storage we suggest cryogenic tubes with external thread as during thawing and homogenization some pressure may build up in the tube, resulting in liquid spraying out when releasing the caps.*

1. Add 6-10 volumes of TRIzol reagent to 1 volume of iRBCs. Close the cap tightly and shake/vortex for 10-30 s until no visible clumps are seen and mixture is homogenous. Thorough mixing in essential for good yields and proper RNA separation. Incubate for 5 min for nucleoproteins to completely dissociate.

- *Tip:*

*As little as 6 volumes of TRIzol can be added to reduce costs and storage space with minor reduction in yield. TRIzol can be also added to samples stored in RNA/DNA Shield or other TRIzol-compatible reagents. We do not recommend any lesser volume of TRIzol as it may result in higher homogenate viscosity, lower yields and possible protein or gDNA contamination.*

- **STOP POINT:** Samples homogenized in TRIzol can be stored for the maximum periods described below:

-80 ^o^C - long term storage

-20 ^o^C - 3 months

4 ^o^C - 15 days

21 ^o^C - 1-3 days (possible mild degradation and RIN drop)

37 ^o^C - storage not recommended at this temperature

- *Tip:*

*TRIzol alone can be used as RNA preserving agent for the periods of time stated above without the need for using additional reagents.*

**SECTION 2 - EXTRACTION**

- TIMING: 20 min – 3 hours (depending on the number of samples)
- TEMPERATURE: RT, 4 ^o^C (if chloroform is used)

**Background**

TRIzol reagent is an acid-guanidinium-phenol based reagent designed for the extraction of RNA, DNA and protein from various biological sample inputs[1]. By controlling pH of sample-TRIzol homogenate we can separate RNA, DNA and protein. The acidic pH allows to separate RNA from DNA and protein, while a basic pH will cause RNA and DNA to be isolated together[2,3]. The guanidinium salt is a chaotropic agent which denatures proteins and phenol is an organic compound also used to extract nucleic acids and proteins[4].

We have tested two variations of the extraction protocol, one with and another without chloroform supplementation. We observed slight increase in yield when using chloroform and recommend using it for high volume samples due to reduced total volume of the aqueous phase. As a result, this will require fewer number of column reloads during RNA binding steps. Extraction without chloroform is recommended for high-throughput work and low volume samples (<100 μl of iRBCs). If not using chloroform start from **Step 8**.

**Workspace setup**

- Pre-chill centrifuge with swing bucket rotor to 4 ^o^C
- Clean all equipment, fume hood, workstations and bench areas with RNase eliminating reagents (e.g. RNaseZap).
- Use only nuclease-free PCR-grade water, labware and reagents
- Change gloves frequently during the protocol to avoid contamination of samples with RNases.
- **CRITICAL:** Working with frozen material.

Thaw samples fast in 60 ^o^C water bath (1-2 min or until no ice present). Alternatively, samples can be defrosted by leaving them on ice. Once thawed, samples must be kept on ice. Do not re-freeze thawed samples.

**Protocol**

OPTION WITH CHLOROFORM (perform steps at 4 ^o^C / on ice)

1. Add 1 volume of chloroform for every 5 volumes of TRIzol used (e.g. 1 ml Chloroform to 5 ml TRIzol).
2. Shake/vortex briefly. Let the mixture sit on ice for 5 min.

- *Tip:*

*To avoid formation of clumps, it is important to add TRIzol to iRBCs and not in the opposite order.*

- *Tip:*

*Always mix samples with TRIzol by shaking/vortexing, not pipetting. Pipetting will clog the tips and result in decreased yield.*

- *Tip:*

*Do not use polystyrene pipettes when working with chloroform. Use polypropylene pipettes instead.*

1. Centrifuge at 4500g for 15 min at 4 ^o^C. Use brake 0 to avoid disturbing separated phases.
2. Gently remove tubes from the centrifuge and place them on ice making sure not to disturb the phases.
3. Transfer upper aqueous phase to a fresh RNase-free tube. Make sure not to aspirate any interphase or lower, organic phase. Proceed to **Step 10**.

- *Tip:*

*Aspirated lower phase may clog silica column, resulting in drop in yield and high phenol contamination in subsequent steps. Leave behind a small volume of top phase when pipetting.*

OPTION WITHOUT CHLOROFORM (perform steps at RT)

1. Spin samples for 1 min at 16000g at RT to bring all debris down. Use brake 9.
2. Aspirate top phase. Do not over-aspirate any of the bottom debris. Bottom phase may be difficult to see; we recommend working in well-lit environment.

- **STOP POINT:** Aqueous phase may be stored at -80 °C long term.

1. Add 1 volume of 100% ethanol (RT) to 1 volume of aqueous phase. Shake well. Tubes can be left at RT during subsequent steps.
2. Apply the above mixture on Direct-zol RNA Microprep silica column (ZYMO) (or Direct-zol-96 RNA if processing samples in high-throughput), no more than 700 μl at each time. Columns can be reloaded if total volume of the mixture exceeds 700 μl.

- *Tip:*

*We do not recommend reloading microprep columns more than four times (i.e. 2.8 ml total sample volume), as it may result in mechanical damage of the column/plate. Please keep in mind that the binding capacity for microprep columns is 10 µg of RNA. If higher RNA yield is expected then miniprep columns should be used. However, only microprep kit comes in a 96-well format.*

- **CRITICAL:** When using individual extraction columns make sure that the sample mixture doesn’t touch the rim of the column. This may result in significant phenol carryover to the final eluent, resulting in a lower 260/230 nm absorbance ratio. This is not a concern when using 96-well format kit.

1. Spin columns following manufacturer’s instructions.
2. Discard supernatant.

- **CRITICAL:** This protocol isolates total RNA with minimal gDNA contamination, thus DNase treatment is not necessary prior to reverse transcription. However, if removal of gDNA is absolutely required, we do not recommend on-column DNase digestion provided with the kit. As an alternative, follow **Steps 72-74** for DNase treatment after extraction.

1. Add 800 μl of Pre-wash buffer.
2. Spin columns following manufacturer’s instructions.
3. Discard supernatant.
4. Add 800 μl of Wash buffer.
5. Spin columns following manufacturer’s instructions.
6. Discard supernatant.
7. Spin columns following manufacturer’s instructions to dry silica column.
8. Transfer columns to new tubes (preferably polypropylene with low DNA/RNA surface binding).
9. Add 10-20 μl of RNase-free water directly on the silica resin. Incubate for 2 min.

- *Tip:*

*It is recommended to keep samples as concentrated as possible for subsequent amplification steps. This can be achieved by eluting in lower volumes (as low as 10 µl).*

1. Spin columns following manufacturer’s instructions
2. Immediately transfer tubes containing RNA eluent on ice.

- **STOP POINT:** Samples can be stored for several months at -80 °C. If using 96-well plates, make sure to use adhesive aluminum seals and press it tightly to ensure that the plates are fully sealed. Low volume samples evaporate at -80 °C over time and are susceptible to edge effect.

**SECTION 3 - RNA QUANTIFICATION**

- TIMING: 5 – 30 min (depending on the number of samples)
- TEMPERATURE: RT

**Background**

After RNA isolation it is paramount to correctly assess yield and purity of extracted RNA. Absorbance methods (e.g. Nanodrop, Thermo Scientific) cannot distinguish between RNA, DNA and protein efficiently and have low tolerance to other contaminants such as free nucleotides, salts and organic compounds in the sample[5]. In addition, this method is not sensitive enough to detect very low concentrations of DNA and RNA. Fluorescence-based assays (e.g. Qubit, Invitrogen) are highly specific to the nucleic acid of interest and have higher sensitivity of detection. However, fluorescence-based assays cannot be used to determine level of contaminants and impurities in the final eluent[6]. Therefore, we recommend using absorbance readings for our Direct-zol extractions in order to determine if the RNA sample is free of TRIzol and protein contamination and fluorescence-based assay for assessing accurate RNA concentration.

**Workspace setup**

- Equilibrate Qubit RNA Broad Range Kit to RT before use.
- Clean all equipment, fume hood, workstations and bench areas with RNase eliminating reagents (e.g. RNaseZap).
- Use only nuclease-free labware and reagents

**Protocol**

1. Quantify RNA concentration and sample purity using Nanodrop and Qubit RNA Broad Range Kit according to manufacturers’ instructions.

- *Tip:*

*Phenol is a known inhibitor of various polymerases*[7,8]*. We recommend using UV absorbance assessment (e.g. Nanodrop) for any TRIzol-based extractions in order to determine if sample is free of phenol contamination. Samples should have an ideal 260/230 ratio values above 1.8.*

- *Tip:*

*For assessing RNA concentration accurately, we recommend using fluorescence-based methods (e.g. Qubit* RNA Broad Range Kit*) as even tiny amounts of guanidinium thiocyanate carryover will cause erroneous quantification on Nanodrop (See:* ***Anticipated results, Fig 1****). Hence, if possible, both absorbance and fluorescence-based methods should be used.*

**SECTION 4 - REVERSE TRANSCRIPTION**

- TIMING: 3 hours
- TEMPERATURE: Set up on ice

**Background**

In this study we implemented SMART-Seq2 method with several notable modifications such as lower elongation and annealing temperatures, adjusted primer concentrations and optimized initial RNA input. Our cDNA amplification protocol works well with as little as 0.05% ring-stage iRBCs. This is roughly 2000-3000 parasites per microliter of whole blood, which in high transmission areas would be considered an infection much below the threshold of symptomatic malaria[9]. However, it is crucial to note that when estimating minimum parasitemia necessary for accurate transcriptome analyses from field samples, possible high human contamination should be considered. Therefore, a safer margin for minimum parasitemia should be used (*See:* **Fig4** and **Results** section). When using RNA-seq platform please refer to **Step 64 *“Tips”*** for additional guidelines.

**Workspace setup**

- All steps during setting up of the reverse transcription reaction should be performed on ice or cool racks to minimize formation of non-specific products and prevent degradation of reagents and RNA.
- Thaw all reagents on ice and prepare reverse transcription mix freshly just before adding to the sample. Keep all reagents on ice when not in use.
- Use fresh DTT supplied with the Superscript™ II kit. No precipitant should be seen in the tube after vortexing.
- Prepare several small aliquots of working concentration dNTP mix (10 mM) and primers (see **Step 33**). Aliquots can be stored in -20 ^o^C.
- Preheat thermal cycler to 72 ^o^C. Lid should be also preheated to 105 ^o^C.

**Protocol**

1. After measuring RNA concentration normalize all samples to 50 ng/µl in a PCR plate.

- *Tip:*

*We recommend using 250 ng of total RNA for reverse transcription. Although, as little as 10 ng can be used, higher RNA input is recommended for low parasitemia samples (See:* **Fig3** and ***Results*** *section in the paper).*

1. Run 1 µl of normalized RNA sample on Agilent RNA 6000 Nano chip according to the manufacturer’s protocol.

- *Tip*

*Agilent microfluidic systems can be used to detect extreme RNA degradation, where most of degraded RNA migrate as short fragments and electropherogram pattern is heavily shifted towards lower marker. There is no clear consensus regarding RIN cut-off number below which samples should not be used for sequencing analyses. However, aim should be placed on working with most intact RNA. As shown in our data, our protocol can generate RNA with RIN approaching 10 for high parasitemia samples and RNA obtained from older parasites (e.g. trophozoites). However, when using the same protocol for young stage parasites or low parasitemia, RIN can drop in value, not necessarily due to sample degradation but due to the limitation of Agilent algorithms or other additional factors.*

*Hence, we recommend that RIN values for RNA extracted from lab cultures should be higher than 7. For high parasitemia and late parasite stages RIN should be approaching 10 (See*: **Fig 1a** and **Fig 2b** in **Results** section*)*

1. Prepare the following primer mix per sample for annealing step:

| **Reagent** | **Final concentration** | **Volume (µl)** |
| --- | --- | --- |
| dNTP mix (10 mM) | 1 mM | 2 |
| Oligo-dT_30_VN (10 µM) | 1 µM | 2 |
| Total volume |  | 4 |

- - *Tip:*

*Prepare one non-template control (water) and one positive control when setting up a new RT reaction and use it for subsequent amplification steps.*

- - *Tip:*

*Always prepare one additional reaction per 20 samples to account for pipetting errors*

1. Add 4 µl of primer mix (from **Step 28**) to 5 µl of pre-normalized RNA sample (from **Step 26**), to make total volume of 9 µl. Seal plate well with adhesive PCR film using film applicator and vortex 5 s.
2. Spin plate at 700g for 10 s at RT.
3. Place the plate in preheated thermal cycler and incubate samples at 72 ^o^C for 3 min.
4. Immediately place the plate back on ice and incubate for 5 min. Spin the plate at 700g for 10 s at RT to bring down any condensation. Put the plate back on ice.
5. Prepare reverse transcription master mix on ice:

| **Reagent** | **Final concentration** | **Volume (µl)** | |
| --- | --- | --- | --- |
| SuperScript II reverse transcriptase (SSII) | 100 U | | 1 |
| RNaseOUT | 10 U | | 0.5 |
| SuperScript II first strand buffer (5x) | 1x | | 4 |
| DTT (0.1 M) | 5 mM | | 1 |
| Betaine (5 M) | 1 M | | 4 |
| MgCl2 (1 M) | 6 mM | | 0.12 |
| LNA-TSO (100 µM) | 1 µM | | 0.2 |
| Nuclease-free water | - | | 0.18 |
| Final volume | - | | 11 |

1. Add 11 µl of the master mix (**Step 33**) to 9 µl of RNA-primer mix (**Step 32**) to obtain a final reaction volume of 20 µl. Mix gently by pipetting.
2. Seal plate with adhesive PCR film and spin at 700g for 10 s at RT.
3. Incubate the reaction plate in a thermal cycler under the following conditions with the lid pre-heated to 105 ^o^C:

| **Number of cycles** | **Temp** | **Time** |
| --- | --- | --- |
| 1 | 42 °C | 90 min |
| 10 | 50 °C | 2 min |
|  | 42 °C | 2 min |
| 1 | 70 °C | 15 min |
|  | 4 °C | hold |

1. Use 5 µl of reverse transcription product (i.e. first strand cDNA) obtained from **Step 36** for subsequent amplification.

- **STOP POINT:** Reverse transcription product can be stored frozen at -80 °C for several months.

**SECTION 5 - SECOND STRAND SYNTHESIS AND cDNA AMPLIFICATION**

- TIMING: 2 hours
- TEMPERATURE: set up on ice

**Background**

High proofreading activity of KAPA HiFi HotStart polymerase results in improved accuracy of amplification. Moreover, its increased affinity to DNA enables more efficient amplification of long and difficult targets (such as AT-rich transcripts). HotStart technology reduces redundant amplification products resulting from non-specific priming events during long reaction setup, which makes it particularly high-throughput and automation-friendly.

**Workspace setup**

- Thaw all reagents on ice and prepare KAPA PCR mix just before adding to the sample.
- *Tip:*

*If additional depletions of unwanted transcripts (e.g. hemoglobin) is necessary please refer to* ***Steps 75-90*** *for in vitro CRISPR-Cas9 based depletion.*

1. Prepare KAPA HiFi HotStart PCR (1x) master mix as follows:

| **Reagent** | **Final concentration** | **Volume (µl)** |
| --- | --- | --- |
| KAPA HiFi HotStart Ready Mix (2x) | 1x | 12.5 |
| ISPCR oligo (100 µM) | 3 µM | 0.75 |
| Nuclease-free water |  | 6.75 |
| Final volume |  | 20 |

- *Tip*

*Prepare one positive control and non-template control (water control) using 5* µl *of samples from reverse transcription steps.*

- *Tip*

*Always prepare one additional reaction per 20 samples to account for pipetting errors.*

1. Add 5 µl of unpurified reverse transcription product (**Step 37**) to 20 µl of master mix (**Step 38**) to obtain final reaction volume of 25 µl. Mix by pipetting.
2. Seal plate with adhesive PCR film and spin at 700g for 10 s at RT.
3. Incubate the reaction plate in a thermal cycler under the following conditions with the lid pre-heated to 105 ^o^C:

| **Number of cycles** |  | **Temp** | **Time** |
| --- | --- | --- | --- |
| 1 |  | 98 °C | 3 min |
| 19 |  | 98 °C | 20 sec |
|  |  | 56 °C 64 °C | 15 sec  6 min |
| 1 |  | 64 °C | 5 min |
|  |  | 4 °C | hold |

- **CRITICAL:** We have experimentally adjusted the annealing and extension temperatures for AT-rich *P. falciparum* sequences. However, when working with other organisms annealing and extension temperatures should be re-optimized if necessary.
- **STOP POINT:** KAPA PCR product can be stored at 4 °C for one day or at -20 °C or -80 °C for several months.

**SECTION 5 - PURIFICATION OF AMPLIFIED cDNA**

- TIMING: 45 min
- TEMPERATURE: RT

**Background**

Purification of amplified cDNA can be performed using magnetic beads (e.g. Ampure XP, Beckman Coulter) or column-based DNA purifications kits (e.g. MinElute PCR purification Kit, Qiagen). Typically, column-based purification has a low-throughput whereas beads-based approach is easily compatible with medium and high-throughput sample processing using 96-well magnetic plate (e.g. Alpaqua Magnum FLX) or liquid handling robotic systems. We successfully adopted the magnetic beads-based purification to Hamilton Nimbus4 platform and created a fully walk-away protocol.

**Workspace setup**

- Before proceeding with purification, equilibrate magnetic beads to RT.
- Prepare fresh 80 % (v/v) analytical grade ethanol.
- Consult Ampure XP magnetic beads manufacturer’s instructions before commencing the protocol.
- Mix beads well to ensure complete homogeneous resuspension of all beads.

**Protocol**

1. Add 45 µl of magnetic beads to PCR amplicon from **Step 41** (ratio 1.8 : 1) and transfer entire content to 96-well round-bottom plate.
2. Mix gently by pipetting at least 10 times or until the solution is homogenous in color. Try to avoid intense pipetting as it will cause foaming.
3. Incubate the mixture at RT for 5 min.
4. Place the plate on magnetic stand for 3 min or until solution is clear (depending on magnet strength)

- *Tip:*

*In this study we used Magnum FLX stand (Alpaqua) for purification as it can accommodate very low elution volumes and has a strong magnet which reduces waiting times and beads disturbance during aspiration. Additionally, Magnum FLX stand’s spring-cushion technology makes it particularly suitable for automation.*

1. With the plate still on the magnet, aspirate clear supernatant slowly making sure that no beads are drawn.

- *Tip*

*Beads can be easily disturbed at this step so we recommend leaving 5* µl *of supernatant behind.*

1. Wash beads with 200 µl of 80% ethanol. Incubate for 30 seconds followed by aspiration of ethanol.

- *Tip:*

*At this stage beads are not easily disturbed. Hence, higher pipetting speeds can be applied.*

- *Tip:*

*Use freshly prepared 80 % ethanol instead of recommended 70%. This will account for evaporation and hygroscopic nature of ethanol, particularly important during robotic handling.*

1. Repeat **Step 47** one more time.
2. Using fresh tips remove any leftover ethanol completely from all samples.
3. Leave the plate at RT for 5 min to dry the beads of any remaining ethanol.

- *Tip*

*Beads should change appearance from glossy dark brow to matt brown. Do not over-dry beads (seen as cracks on the beads pellet) as it will result in long DNA fragment retention and loss of final yield.*

1. Add the required volume of EB (10 mM Tris-Cl, pH 8.5). Take the plate off the magnet and incubate at RT for 3 minutes.

- *Tip*

*Eluting samples in low volumes of buffer will keep them concentrated for subsequent steps and storage (recommended elution volume is 20 µl)*

1. Mix beads with EB by pipetting until all beads are in solution and the mixture is homogenous.
2. Incubate the mixture for 5 min.
3. Place the plate back on the magnet for 1 min or until solution clears.
4. Aspirate cleared solution slowly and transfer it to a fresh 96-well plate.

- *Tip*

*When pipetting, aspirate slowly and leave few microliters (~1-1.5 µl) behind to avoid aspiration of beads.*

- **STOP POINT:** Purified cDNA can be stored at -20 °C or -80 °C long term.

1. Measure the concentration of purified cDNA using fluorescence-based kits such as Qubit dsDNA Broad Range (Invitrogen) or Quant-IT Broad Range dsDNA (Invitrogen) for high-throughput studies.
2. If available, run 1 µl of the sample on Bioanalyzer DNA 12000 chip to observe the size distribution of the amplified product. (*See:* **Anticipated results, Fig 2**).

- *Tip:*

*Bioanalyzer DNA 12000 is sensitive to overloading. Load only 20 to 50 ng of cDNA per well.*

**SECTION 6 - LIBRARY PREPARATION FOR SEQUENCING**

- TIMING: 2-3 hours
- TEMPERATURE: RT

**Background**

Library preparation protocols will differ depending on the kit used. For our applications we have used the Illumina Nextera XT Library Prep Kit optimized for small genomes, PCR amplicons, plasmids and cDNA. Follow **Step 58 – Step 64** for library preparation if using above mentioned kit.

**Protocol**

1. Take 5 µl of each sample from **Step 55** and dilute them to 1 ng/µl using EB. Mix each sample well by pipetting or vortexing followed by spinning the plate at 700g for 10s at RT.
2. Measure the concentrations of diluted samples with high sensitivity fluorescence-based assay (e.g. Qubit High Sensitivity dsDNA Kit, Invitrogen)
3. Take 10 µl of each normalized sample from **step 58** and dilute them to 0.2 ng/µl using EB.
4. Follow manufacturer’s instructions for library preparation using the Nextera XT Library Preparation Kit (available at [www.illumina.com](http://www.illumina.com)).

- *Tip*

*Please consult Illumina guidelines for index primer compatibility when preparing libraries for samples which will be multiplexed on one lane.*

1. After PCR amplification of index adapter-ligated Illumina library, purify PCR product using Ampure XP magnetic beads following **Step 42** – **Step 55** with the beads to sample volume adjusted accordingly (ratio 0.6:1, v/v).

- **CRITICAL:** Use 0.6:1 (v/v) beads to sample ratio to minimize primer-dimer carryover and select library fragments larger than 300bp (e.g. 30 µl beads to 50 µl PCR product). Failing to do so will result in excessive amount of short library fragments which will cause loss of sequencing reads and reading through adapters during sequencing (*See:* **Anticipated results, Fig 3c**).

1. To check library size distribution run 1 µl of each purified library on Agilent High Sensitivity DNA chip. Note down average fragment size as it will be needed for pooling calculations in subsequent steps (*See:* **Anticipated results, Fig 3**).
2. Prepare denatured libraries and pool them in equimolar ratios according to Nextera XT pooling guidelines for each type of sequencing platform used.

- *Tip:*

*Equimolar pooling of libraries is crucial for differential gene expression analysis. We recommend using pooling methods based on quantitative PCR (i.e. manual) instead of beads-based methods due to higher consistency and accuracy. Quantitative PCR based methods assess adapter incorporation into libraries.*

- *Tip:*

*Quality control, pooling and denaturing of libraries can be time consuming and prone to handling errors. Thus, it may be worth considering outsourcing these final steps to the sequencing facility.*

- *Tip:*

*Maximum number of samples pooled on each lane will depend on the sequencing platform used, library quality, nature of the experiment and the density of parasitized RBCs. It will have to be assessed experimentally. Our 18S-Pf/18S-Hs ribosomal peak-height ratio derived from Bioanalyzer results can help in assessment of how many samples could be multiplexed per lane in order to obtain satisfactory parasite transcriptome coverage. As shown, in order to obtain 16% of Plasmodium unique reads, the 18S-Pf/18S-Hs ratio value should be 0.4 or above (See:* ***Fig 4d****). As a safety margin we recommend to aim for at least 3x10^6^ unique Plasmodium reads output from each sequencing experiment. Based on the data derived from the analysis of field specimens, samples reach 18S-Pf/18S-Hs values of 0.4 at a median parasitemia of 38000 parasites/µl*.

*For more information regarding optimal numbers of samples for pooling, refer to* ***Results*** *sections in the paper.*

**SECTION 7 - SAMPLE PREPARATION FOR MICROARRAY LABELING**

- TIMING: 2 hours
- TEMPERATURE: on ice, RT

**Background**

As far as readout platform is concerned, low parasitemia clinical samples gave us much better correlations to the reference IDC transcriptome when microarray platform was used instead of RNA-seq (*See*: **Fig4b, Fig4c** and **Results** section). This suggests that despite the advantages of RNA-seq such as sensitive transcript detection, broader dynamic range, reproducibility, low batch effects, less technical noise, splice variant detection, etc., microarrays are still very robust when working with mixed-species samples[10–12]. This is possibly due to parasite cDNA specifically hybridizing to the parasite target oligos thus reducing signal from contaminating human cDNA. However, when dealing with low parasitemia samples on RNA-seq platform, there is always a possibility to opt for deeper sequencing by reducing number of samples multiplexed on a single lane, thus increasing coverage of parasite specific transcripts. The current limitation with this approach is higher cost.

Amplified samples from **Step 55** can be also analyzed using microarray hybridization platform[13]. Here, we present initial steps necessary for preparation of modified cDNA for two color spotted oligonucleotide microarrays.

KAPA HiFi polymerase is unable to incorporate aa-dUTPs during cDNA amplification. This is however necessary for Cy dye labeling of samples, thus requiring an additional round of amplification using a different polymerase that tolerates presence of aa-dUTPs.

**Workspace setup**

- All steps should be performed on ice or cool racks to minimize formation of non-specific products and prevent degradation of reagents.
- Thaw all reagents on ice and prepare master mix just before adding to the sample. Keep all reagents on ice when not in use.
- Always mix and spin samples before starting the protocol.

**Protocol**

1. Prepare samples from **Step 55** by diluting them to 20 ng/µl.
2. Prepare a mix of aa-dNTPs as follows:

| **Reagent** | **Final concentration** | **Volume (µl)** |
| --- | --- | --- |
| dATP (100 mM) | 30 mM | 30 |
| dCTP (100 mM) | 15 mM | 15 |
| dGTP (100 mM) | 15 mM | 15 |
| dTTP (100 mM) | 15 mM | 15 |
| aa-dUTP (100 mM) | 15 mM | 15 |
| Nuclease free water | - | 10 |
| Total | - | 100 |

1. Prepare *Taq* polymerase PCR master mix as follows:

| **Reagent** | **Final concentration** | **Volume (µl)** |
| --- | --- | --- |
| 10x NEB *Taq* buffer | 1x | 5 |
| ISPCR oligo (100 µM ) | 3 µM | 1.5 |
| aa-dNTP mix | - | 0.75 |
| *Taq* Polymerase (5 U/µl) | 0.1 U/µl | 1 |
| Nuclease-free water | - | 36.75 |
| Total | - | 45 |

- *Tip*

*Always prepare one additional reaction per 20 samples to account for pipetting errors*

1. Add 45 µl of master mix (**Step 67**) to 5 µl of sample (100 ng total cDNA) (**Step 65**) to obtain final reaction volume of 50 µl. Mix by pipetting.
2. Seal the plate with adhesive PCR film and spin at 700g for 10 s at RT.
3. Incubate the reaction in thermal cycler with lid heated to 105°C using previously described conditions[14]:

| **Number of cycles** |  | **Temperature** | **Time** |
| --- | --- | --- | --- |
| 1 |  | 95 °C | 5 min |
| 1 |  | 60 °C | 1 min |
| 1 |  | 68 °C | 10 min |
| 19 |  | 95 °C | 30 sec |
|  |  | 60 °C 68 °C | 30 sec  5 min |
| 1 |  | 72 °C | 5 min |
|  |  | 4 °C | hold |

1. Purify samples using AMPure XP magnetic beads following **Step 42** – **Step 55** remembering to adjust beads to sample volume accordingly (1.8:1, v/v).

- *Tip*

*Elute samples in low volumes of EB to keep them concentrated for subsequent dye labeling steps (recommended 17 µl)*

- **STOP POINT**: Purified cDNA can be stored at -20 °C or -80 °C for several months.
- *Tip:*

*cDNA with aa-dUTPs incorporated into its sequence can be labeled with Cy-dyes and hybridized on various microarray platforms.*

**ADDITIONAL PROTOCOL EXPANSIONS**

**SECTION 8 - DNase TREATMENT**

- TIMING: 45 min
- TEMPERATURE: on ice

**Background**

We did not detect much of gDNA presence in the final product of this pipeline as suggested by low reads numbers mapping to intergenic regions**.** Several factors could contribute to this; for example, efficient initial leucocyte depletion or use of oligo-dT primers instead of random primers during reverse transcription will reduce gDNA contamination. Additionally, Direct-zol RNA extraction was designed to effectively separate gDNA from the RNA eluent. Nevertheless, for assays/studies very sensitive to gDNA contamination, following steps can be performed in order to remove residual gDNA from extracted RNA.

**Workspace setup**

- All steps should be performed on ice or cool racks to prevent degradation of samples and/or reagents.
- Thaw all reagents on ice and prepare master mix just before adding to the samples. Keep all reagents on ice when not in use.

**Protocol**

1. Aliquot total RNA (**Step 24**) amount necessary for further assays. Remember to account for additional reagents when calculating final volume.
2. Add the following reagents for DNase treatment of RNA:

| **Reagent** | **Final concentration** | **Volume (µl)** |
| --- | --- | --- |
| RNA | - | 5 |
| DTT (100 mM) | 2.6 mM | 0.15 |
| MgCl2 (50 mM) | 3 mM | 0.35 |
| HL-dsDNase (2 U/µl) | 0.1 U/ µl | 0.29 |

- *Tip*

*Final DTT concentration should be at least 1 mM to ensure complete deactivation of DNase in subsequent steps.*

- *Tip*

*If higher or lower volumes of RNA need to be treated, add 0.1 U HL-dsDNase (ArcticZymes) accordingly per one 1 µl of RNA preparation. Mix well by pipetting.*

1. Incubate sample-DNase mix in a thermocycler with a pre-heated lid under the following conditions:

| **Number of cycles** |  | **Temperature** | **Time** |
| --- | --- | --- | --- |
| 1 |  | 25 °C | 15 min |
| 1 |  | 55 °C | 10 min |

- *Tip*

*Further purification is not required after treatment. Samples can be used directly for quantification* ***(Step 25)*** *and reverse transcription* ***(Step 26).***

**SECTION 9 - CRISPR-Cas9 *in vitro* HEMOGLOBIN TRANSCRIPTS DEPLETION**

- TIMING: 2 hours
- TEMPERATURE: on ice

**Background**

Our RNA-sequencing data revealed that ~ 70% of the reads mapping to human genome belong to the hemoglobin gene family (majorly HBA1, HBA2 and HBB). In order to selectively deplete HBA1, HBA2 and HBB transcripts, we treated amplified cDNA with sequence-specific guide RNA (gRNA) and Cas9 nuclease to perform *in vitro* selective depletion of targeted sequences.

1. Perform **Step 38** – **Step 57** using this modified PCR conditions:

| **Number of cycles** |  | **Temperature** | **Time** |
| --- | --- | --- | --- |
| 1 |  | 98°C | 3 min |
| 5 |  | 98°C | 20 sec |
|  |  | 56°C 64°C | 15 sec  6 min |
| 1 |  | 64°C | 5 min |
|  |  | 4°C | hold |

1. Measure samples using fluorescence-based kit such as Qubit dsDNA High Sensitivity or Quant-IT dsDNA high-sensitivity for high-throughput studies.
2. Calculate the molarity of target sequences in a given amount of cDNA using the following formula:

**Molarity (mol/L) = Mass (g) / (total solution volume (L) x molecular weight (g/mol))**

1. Calculate the amount of each gRNA and Cas9 (1000:1 ratio) to use per reaction to obtain the desired ratio of gRNA-Cas9 to target sequences. Below is an example of calculation for cDNA at 50 ng/µl concentration. Since the HBB transcript size is 3932 bases its molarity will be 19.27 nM (calculated using the above formula).

| **HBB (1000 : 1)** | **Volume (µl)** | **Final concentration** |
| --- | --- | --- |
| **cDNA (50 ng/µl – 19.27 nM)** | 1 | 0.96 nM (final HBB molarity in 20 µl) |
| **10X Cas9 buffer** | 2 | 1x |
| **gRNA (20 µM )** | 0.96 | 960 nM |
| **Cas9 (20 µM)** | 0.96 | 960 nM |
| **H2O** | 15.08 | - |
| **Total** | 20 | - |

1. Mix the required amount of gRNA and Cas9 in Cas9 buffer in a PCR tube.
2. Incubate the mixture at 37 ^o^C for 10 min in a thermocycler to allow the formation of gRNA-Cas9 complex.
3. Add the desired amount of sample DNA to the mixture to the final volume of 20 µl.
4. Incubate the mixture at 37 ^o^C for 2 hours.
5. Inactivate Cas9 by adding 1 µl of Proteinase K (20 mg/ml) to the mixture, and incubate at 37 ^o^C for an additional 15 min.
6. Purify the CRISPR-Cas9 treated cDNA following **Steps** **42-55** and adjusting beads to sample volume at 1.8:1 (v/v) ratio.
7. Elute samples in 11.75 µl of EB (10 mM Tris-Cl, pH 8.5) and use the entire volume for subsequent amplification steps.
8. Prepare PCR master mix as stated below:

| **Reagent** | **Final concentration** | **Volume (µl)** |
| --- | --- | --- |
| **KAPA HiFi HotStart Ready Mix (2x)** | 1x | 12.5 |
| **ISPCR oligo (100 µM)** | 3 µM | 0.75 |
| **Final volume** |  | 13.25 |

1. Add entire volume of sample from **Step 85** (11.75 µl) to 13.25 µl of master mix (**Step 86**) to obtain final reaction volume of 25 µl. Mix by pipetting.
2. Seal the plate with an adhesive PCR seal and spin briefly.
3. Incubate the reaction plate in a thermal cycler with lid heated to 105 °C using the conditions below:

| **Number of cycles** |  | **Temp** | **Time** |
| --- | --- | --- | --- |
| 1 |  | 98 °C | 3 min |
| 14 |  | 98 °C | 20 sec |
|  |  | 56 °C 64 °C | 15 sec  6 min |
| 1 |  | 64 °C | 5 min |
|  |  | 4 °C | hold |

1. Purify the mixture by following **Steps 42-55** using beads to sample volume at 1.8:1 (v/v) ratio.

- **STOP POINT:** KAPA PCR product can be stored at 4 °C for one day or at -20 °C or -80 °C for several months.

**SECTION 10 - COMPUTATIONAL ANALYSES**

**Background**

Raw reads obtained from sequencer were first checked for overall quality and trimmed to remove adapters, amplification primers and low quality bases from 3’-ends using TRIMGALORE[15]. HISAT2 aligner was used to perform alignment to the genome[16]. Paired reads with proper orientation mapped to unique locations of genome were considered for counting[17]. Using BEDTools gene specific read counts were calculated[18]. Normalized counts (FPKM and TPM) were then calculated and used for further analysis.

**Protocol**

| **Step** | **Input data** | **Output data** |  | **Tools used** | **Description** |
| --- | --- | --- | --- | --- | --- |
| **A** | Raw sequencing reads | Processed reads |  | Trim Galore | Trimming of reads (adapters and primers) and quality filtering |
| **B** | Processed reads | Aligned reads |  | HISAT2 | Reads alignment to reference genome |
|  |  |  |  |  |  |
| **C** | Aligned reads | Filtered alignment |  | SAMTOOLS | Filtering aligned reads (selecting unique reads) |
| **D** | Filtered alignment | Raw count |  | BEDTools | Counting reads |
| **E** | Raw count | Normalized count |  | Microsoft Excel | Calculating FPKM/TPM |

**SECTION 11 - ANTICIPATED RESULTS**

**a)**


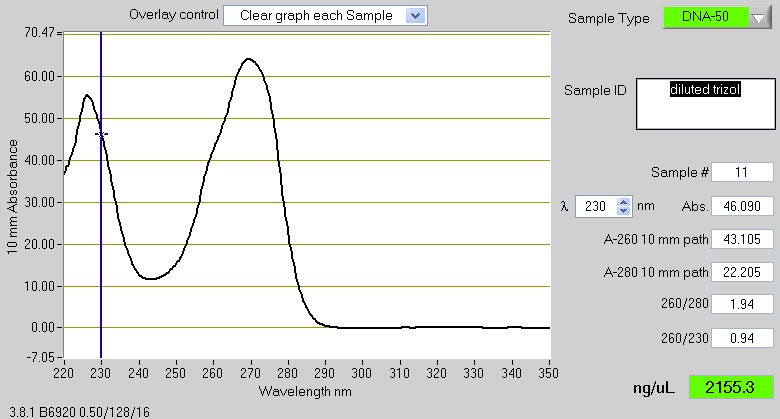


**d)**

**c)**

**b)**


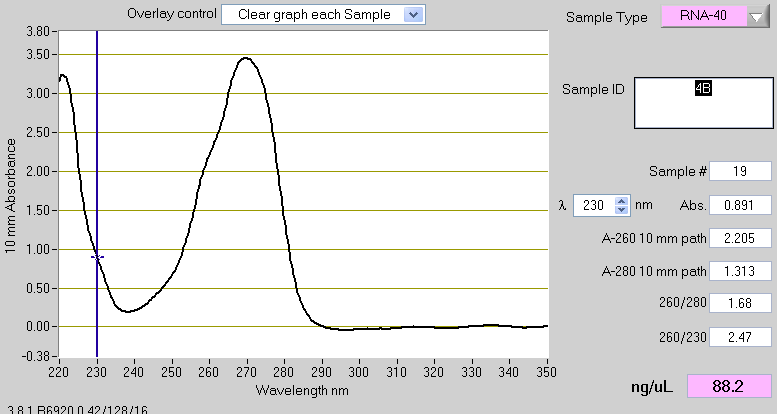

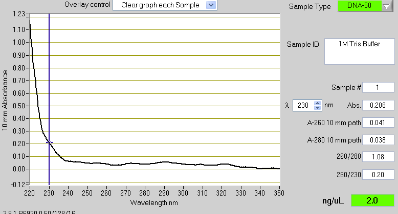

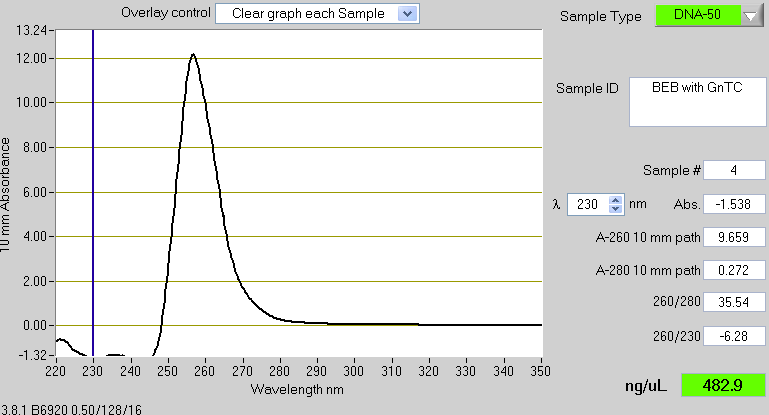


**Fig 1: Nanodrop traces showing absorbance peaks of commonly used RNA extraction reagents and buffers with no DNA or RNA present**. **(a)** TRIzol **(b)** Guanidinium thiocyanate **(c)** 10 mM Tris-HCl **(d)** Acidic phenol. TRIzol and phenol are showing very high absorbance at 220-230 nm and 270 nm wavelengths. Guanidinium thiocyanate shows high absorbance at 260 nm. Even small amounts of this compound present in final RNA/DNA eluents can affect accurate concentration measurements.

**c)**

**b)**

**a)**


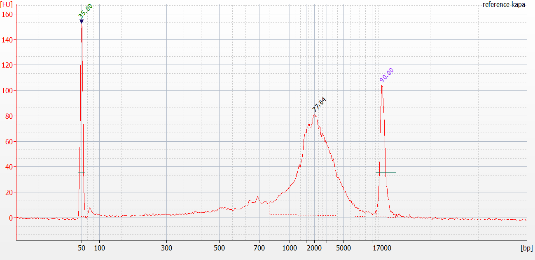

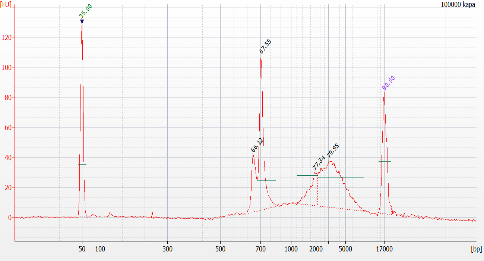

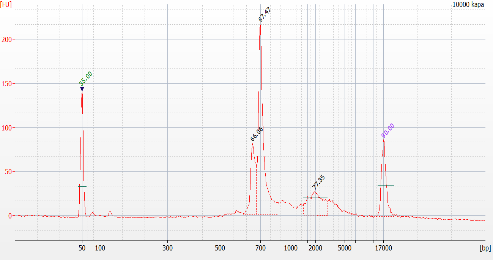


**Fig 2**: **Bioanalyzer electropherograms showing cDNA amplified using KAPA HiFi polymerase.** **a)** Samples with low parasitemia (0.1%) **b)** samples with medium-high parasitemia (1%) **c)** samples with very high parasitemia (10%). With low and medium-high parasitemia (a, b) two distinct haemoglobin peaks (HBB and HBA) can be observed around 700bp. When dealing with undetermined parasitemia, presence or absence of haemoglobin peaks can serve as a potential predictor of unique parasite to human transcripts ratio in the sample (subjective to efficient WBC removal from the sample).

**c)**

**b)**

**a)**


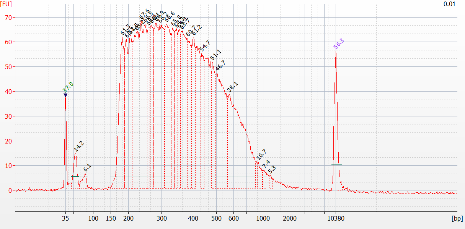

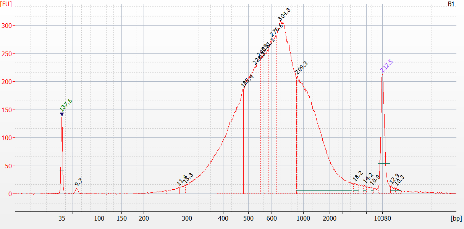

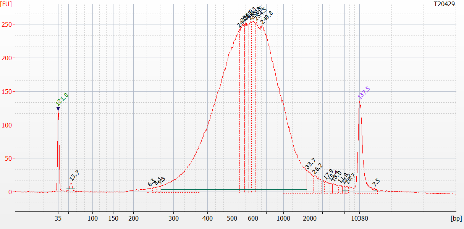


**Fig 3**. **Bioanalyzer DNA High Sensitivity electropherograms showing examples of cDNA libraries prepared with Nextera XT Library Prep Kit.** **(a)(b)** Successfully sequenced libraries with average sizes ranging from 500-700bp for *Plasmodium falciparum* transcripts **(c)** Problematic libraries with sizes averaging at 200-300bp. When using 150bp pair-end sequencing, primer read-through contamination was observed. All library sizes shown include cDNA construct, indices, universal PCR primers and SMART-Seq2 specific primers.

**SECTION 12 - TROUBLESHOOTING**

| **Step** | **Problem** | **Possible reason** | **Solution** |
| --- | --- | --- | --- |
| **Step 1 – Step 9** | Aqueous phase “jellified” | Incorrect ratio of chloroform to TRIzol used | Use only 1:5 Chloroform : TRIzol ratio |
|  | Brownish, not clear aqueous (upper) phase | Free heme or hemoglobin contamination. Incomplete mixing, too short incubation of the homogenate, clumps. Dark brown aqueous phase will normally occur when no chloroform is added. | Vigorously mix RBCs with TRIzol. Mix thoroughly after adding chloroform. Incubate at least 3 min at RT after mixing. |
|  | Clumps in organic (lower) phase | Incomplete homogenization after adding TRIzol | Add TRIzol to the sample and not sample to TRIzol. Mix sample vigorously immediately after adding TRIzol by shaking or vortexing. Do not mix by pipetting. Frozen blood samples will clump faster than fresh ones. More mixing may be required. |
|  | No sharp phase separation | Spin too short and too slow, too high braking speed, incorrect spin temperature. | Spin tubes for at least 5 min at 4500g. Use swing bucket, do not apply brake (Brake 0), pre-chill centrifuge to 4 ^o^C |
|  | Very little upper phase, hard to separate | Low RBCs volume | Use smaller tubes. Apply protocol without chloroform |
|  |  |  |  |
| **Step 10 – Step 24** | Silica column blocked | Over aspiration of proteins and lipids from lower phase | Do not try to aspirate all top phase volume, leave some behind |
|  | Silica powder dislocates | Excessive volume of sample loaded | Do not reload columns more than 4-5 times |
|  | Columns or plates cracked | Excessive centrifugation | Do not reload columns more than 4-5 times |
| **Step 25** | Low 260/230 ratio | Phenol carryover | Make sure not to wet the rim of the column when transferring sample-ethanol mix. Perform extra dry spin after second washing step.  Repurification may be necessary |
|  | Low RNA yields | Low sample volume  Low parasitemia  Insufficient RNA binding to silica    Insufficient homogenization of the sample | If possible work with highly parasitized samples or parasites in late developmental stage.  When working with small volume samples it might be better to skip phase separation with chloroform and procced with no-chloroform protocol.  Make sure to add 1:1 volume of 100% ethanol to aqueous phase.  Mix sample vigorously immediately after adding TRIzol by shaking or vortexing |
|  |  | Possible RNase contamination | Work only using RNase-free consumables. Decontaminate surfaces and equipment before commencing the protocol. Always keep RNA samples on ice. |
| **Step 28 – Step 56** | Low cDNA yield | Presence of inhibitors | Phenol present in TRIzol is known to be a strong inhibitor of reverse transcription and PCR. Make sure that Nanodrop 260/230nm and 260/280 ratio is 1.8 and above.  Always use clean labware and prepare master mix in clean conditions (preferably in dedicated PCR hood) |
|  | Too high cDNA yield.  High cDNA yield in non-template control | Presence of contaminants | Make sure that the reagents are fresh and clean. Always use clean labware and prepare master mix in clean conditions (preferably in dedicated PCR hood) |
| **Step 63** | Small library fragments size | Over-tagmentation  Incorrect beads to sample ratio used during purification step | Make sure to use exactly 1 ng of cDNA for library preparation. Too low input may result in smaller library sizes due to over-tagmentation by Tn5 transposase.  Remeasure concentrations if necessary.  Use 0.6x (v/v) beads to PCR product ratio when purifying final library product. |
|  | Primer dimers present on Bioanalyzer electropherogram | Incorrect beads to sample ratio used during purification step | Use 0.6x (v/v) beads to PCR product ratio when purifying final library product. Note that this differs from 1.8x ratio used initially during cDNA purification. |

**REFERENCES**

1. Chomczynski P, Sacchi N. The single-step method of RNA isolation by acid guanidinium thiocyanate-phenol-chloroform extraction: Twenty-something years on. Nat Protoc. 2006;1:581–5.

2. Perry RP, Kelley DE. The production of ribosomal RNA from high molecular weight precursors. III. Hydrolysis of pre-ribosomal and ribosomal RNA by a 3′-OH specific exoribonuclease. J Mol Biol. Academic Press; 1972;70:265–79.

3. Brawerman G, Mendecki J, Lee SY. A Procedure for the Isolation of Mammalian Messenger Ribonucleic Acid. Biochemistry. 1972;11:637–41.

4. Chirgwin JM, Przybyla AE, MacDonald RJ, Rutter WJ. Isolation of Biologically Active Ribonucleic Acid from Sources Enriched in Ribonuclease. Biochemistry. 1979;18:5294–9.

5. R. Desjardins P, Conklin DS. Microvolume Quantitation of Nucleic Acids. Curr Protoc Mol Biol. Hoboken, NJ, USA: John Wiley & Sons, Inc.; 2011. p. A.3J.1-A.3J.16.

6. Simbolo M, Gottardi M, Corbo V, Fassan M, Mafficini A, Malpeli G, et al. DNA Qualification Workflow for Next Generation Sequencing of Histopathological Samples. PLoS One. Public Library of Science; 2013;8.

7. Schrader C, Schielke A, Ellerbroek L, Johne R. PCR inhibitors - occurrence, properties and removal. J Appl Microbiol. 2012;113:1014–26.

8. Rossen L, Nørskov P, Holmstrøm K, Rasmussen OF. Inhibition of PCR by components of food samples, microbial diagnostic assays and DNA-extraction solutions. Int J Food Microbiol. 1992;17:37–45.

9. Smith T, Killeen G, Lengeler C, Tanner M. Relationships between the outcome of Plasmodium falciparum infection and the intensity of transmission in Africa. Am J Trop Med Hyg. 2004. p. 80–6.

10. Zhao S, Fung-Leung WP, Bittner A, Ngo K, Liu X. Comparison of RNA-Seq and microarray in transcriptome profiling of activated T cells. PLoS One. Public Library of Science; 2014;9.

11. Costa V, Angelini C, De Feis I, Ciccodicola A. Uncovering the Complexity of Transcriptomes with RNA-Seq. J Biomed Biotechnol. Hindawi Publishing Corporation; 2010;2010.

12. Westermann AJ, Gorski SA, Vogel J. Dual RNA-seq of pathogen and host. Nat. Rev. Microbiol. Nature Publishing Group; 2012. p. 618–30.

13. Bozdech Z, Mok S, Gupta AP. DNA Microarray-Based Genome-Wide Analyses of Plasmodium Parasites. 2012. p. 189–211.

14. Rocamora F, Zhu L, Liong KY, Dondorp A, Miotto O, Mok S, et al. Oxidative stress and protein damage responses mediate artemisinin resistance in malaria parasites. PLoS Pathog. Public Library of Science; 2018;14:e1006930.

15. Babraham Bioinformatics - Trim Galore! [Internet]. [cited 2020 May 1]. Available from: https://www.bioinformatics.babraham.ac.uk/projects/trim_galore/

16. Kim D, Paggi JM, Park C, Bennett C, Salzberg SL. Graph-based genome alignment and genotyping with HISAT2 and HISAT-genotype. Nat Biotechnol. Nature Publishing Group; 2019;37:907–15.

17. Li H, Handsaker B, Wysoker A, Fennell T, Ruan J, Homer N, et al. The Sequence Alignment/Map format and SAMtools. Bioinformatics. 2009;25:2078–9.

18. Quinlan AR, Hall IM. BEDTools: A flexible suite of utilities for comparing genomic features. Bioinformatics. 2010;26:841–2.
