# Supplementary material for: A comprehensive RNA handling and transcriptomics guide for high-throughput processing of Plasmodium blood-stage samples
Source: Malar J. 2020 Oct 9;19:363. doi: 10.1186/s12936-020-03436-w (PMC7547485; doi:10.1186/s12936-020-03436-w)
Supplement: Supplementary file 2 — Additional file 2. Supplementary Figures. [file 12936_2020_3436_MOESM2_ESM.pdf]

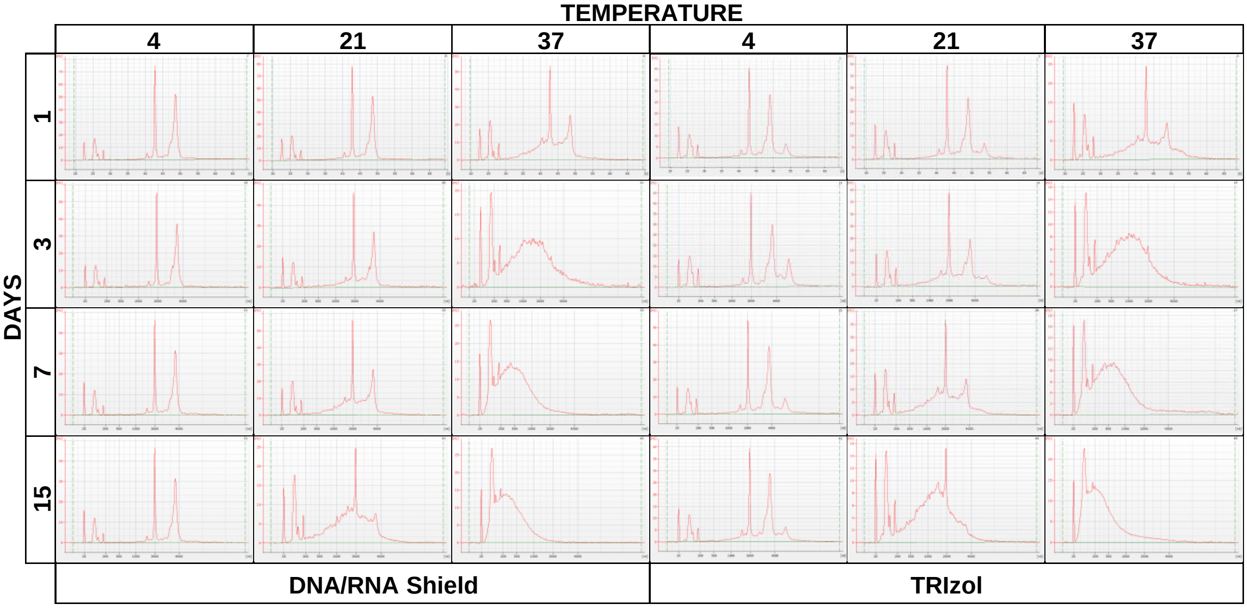

Supplementary Fig S1.
Agilent Bioanalyzer RNA Nano6000 electropherograms showing RNA preservation efficacy in two preserving media across different temperatures and storage times.

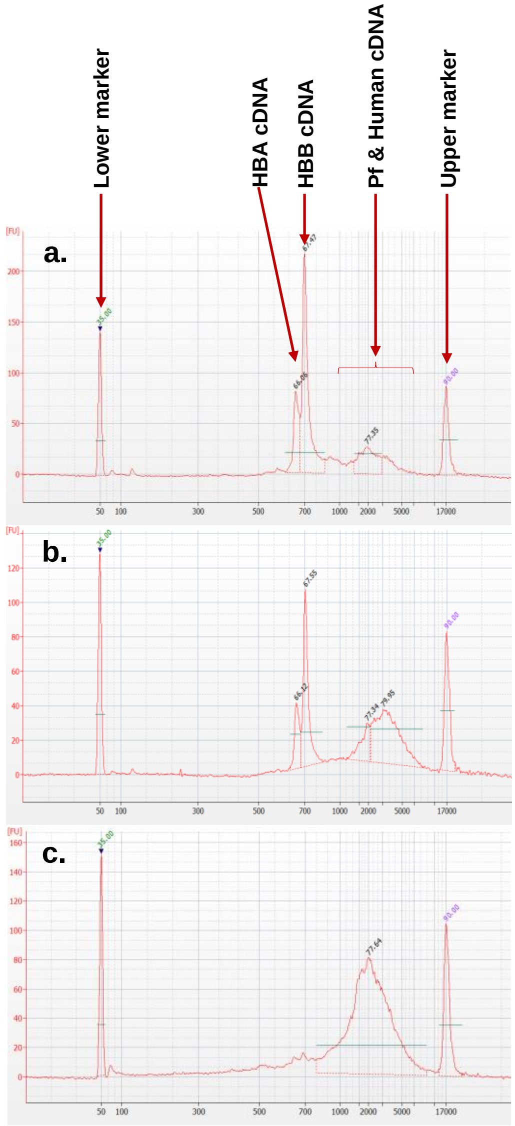

Supplementary Fig S2.
Agilent Bioanalyzer DNA 12000 electropherograms of amplified cDNA. In low and high parasitemia samples (a,b) two distinct haemoglobin peaks can be seen. In very high parasitemia samples (c) haemoglobin peaks disappear almost completely. **(a)** Samples with low parasitemia (0.1%). **(b)** Samples with high parasitemia (1%). **(c)** Samples with very high parasitemia (10%).

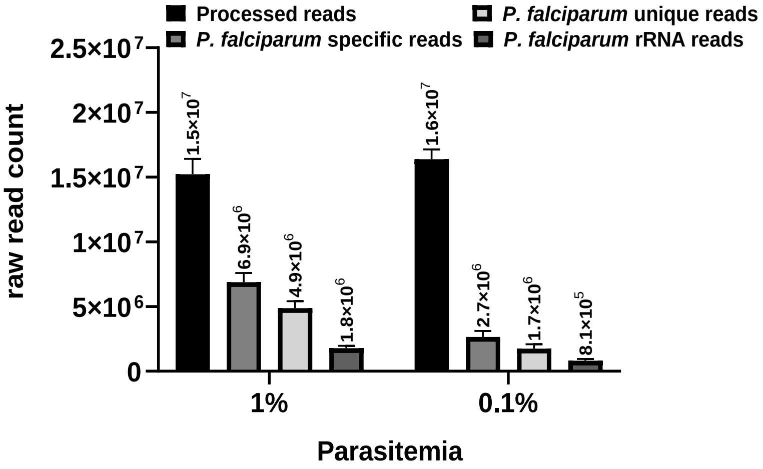

Supplementary Fig S3.
Averaged (n=3) paired-end read counts for 1% and 0.1% parasitemia samples, classified according to mapping specificity, uniqueness and location in the parasite genome. Samples sequenced on Illumina HiSeq 4000 platform with total 24 samples multiplexed per lane (final output 110Gb/lane)

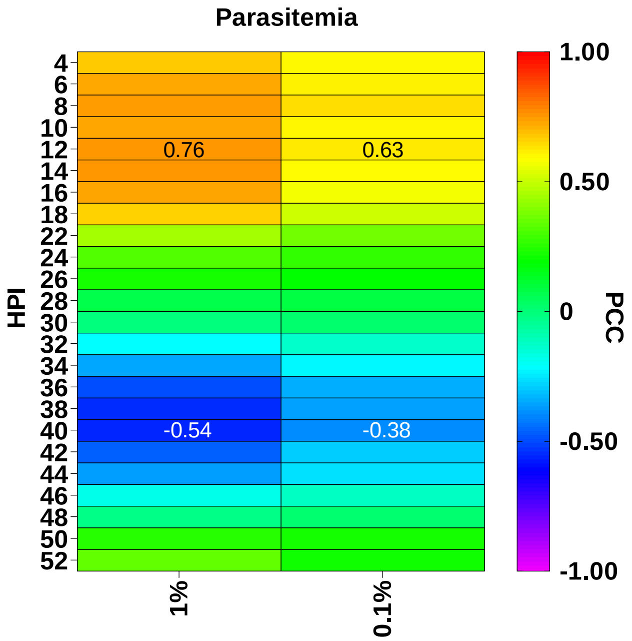

Supplementary Fig S4.
A heatmap of correlation of 1% and 0.1% parasitemia samples (rings, 12 hpi) to 3D7 IDC reference transcriptome based on FPKM values obtained from whole transcriptome RNA sequencing. Samples sequenced on Illumina HiSeq 4000 platform with total 24 samples multiplexed per lane (final output 110Gb/lane). Highest PCC values are indicated. HPI= hours post invasion.

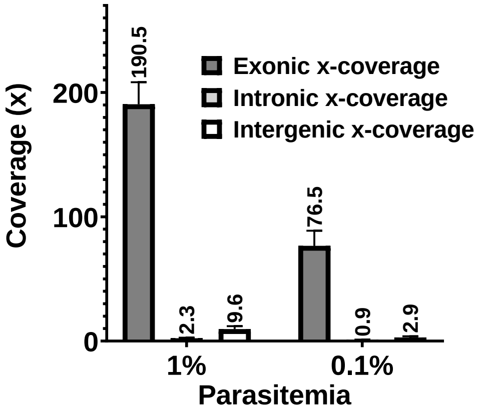

Supplementary Fig S5.
Average x-coverage of different genomic regions in high (1%) and low (0.1%) parasitemia samples.

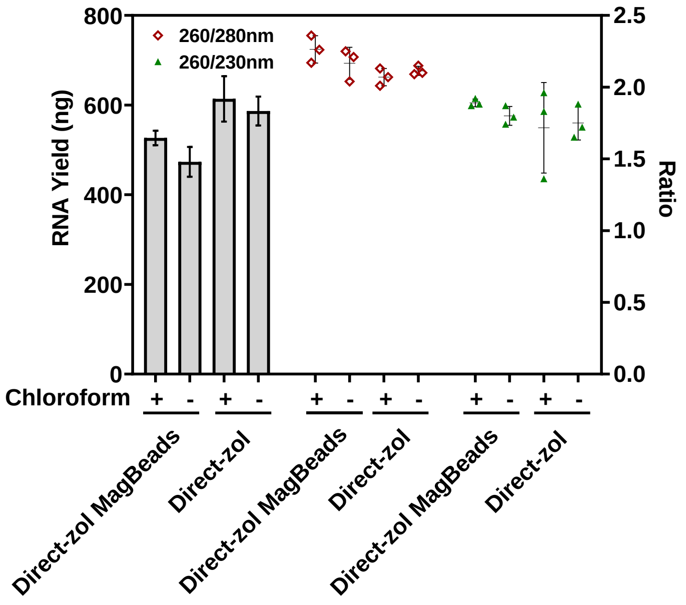

Supplementary Fig S6.
Comparison of Direct-zol MagBeads or Direct-zol RNA extraction methods. Total RNA yields are shown in grey bars. RNA purity was assessed with Nanodrop readings at 230nm, 260nm and 280 nm wavelengths and the ratios are shown. Chloroform supplementation to the TRIzol homogenate is indicated as +/-.

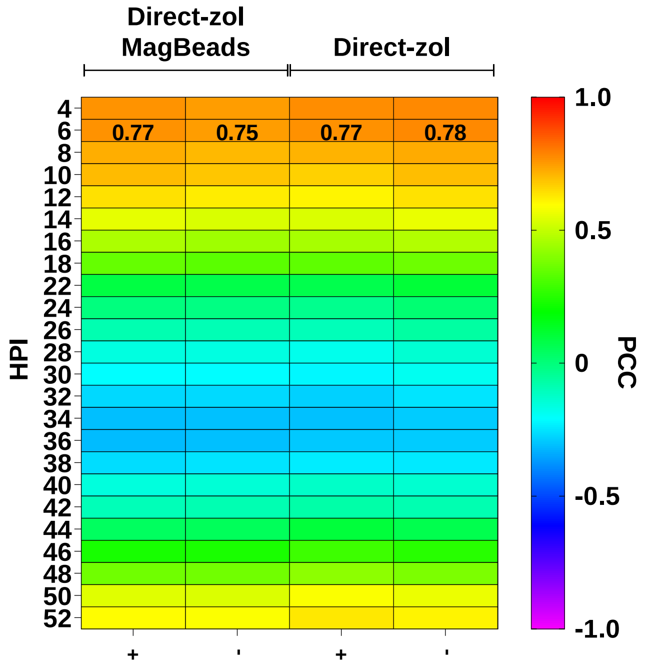

Supplementary Fig S7.
A heatmap showing correlation of samples with RNA extracted using Direct-zol MagBeads or Direct-zol to 3D7 IDC reference transcriptome. Supplementation of chloroform to the lysate is indicated as +/-. Highest PCC values are indicated. HPI= hours post invasion.

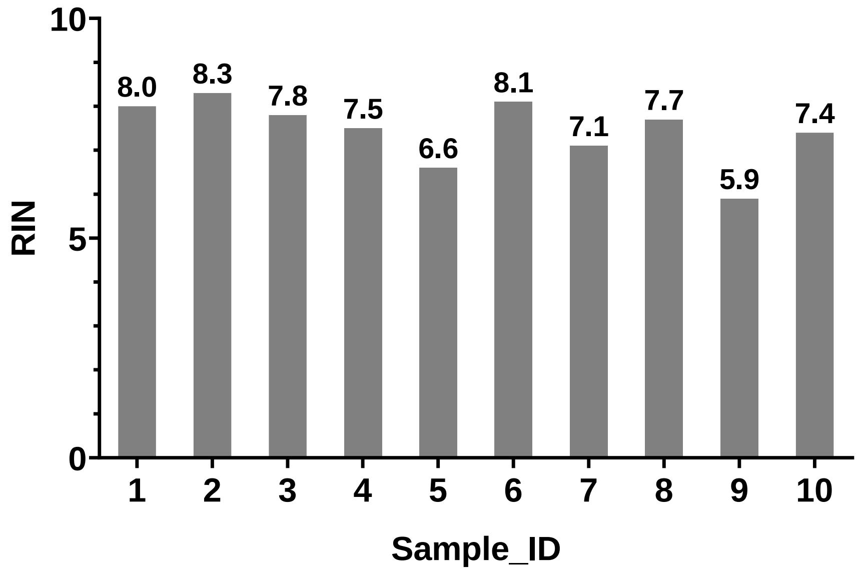

Supplementary Fig S8
RNA integrity measure reflected by RIN from Agilent Bioanalyzer.

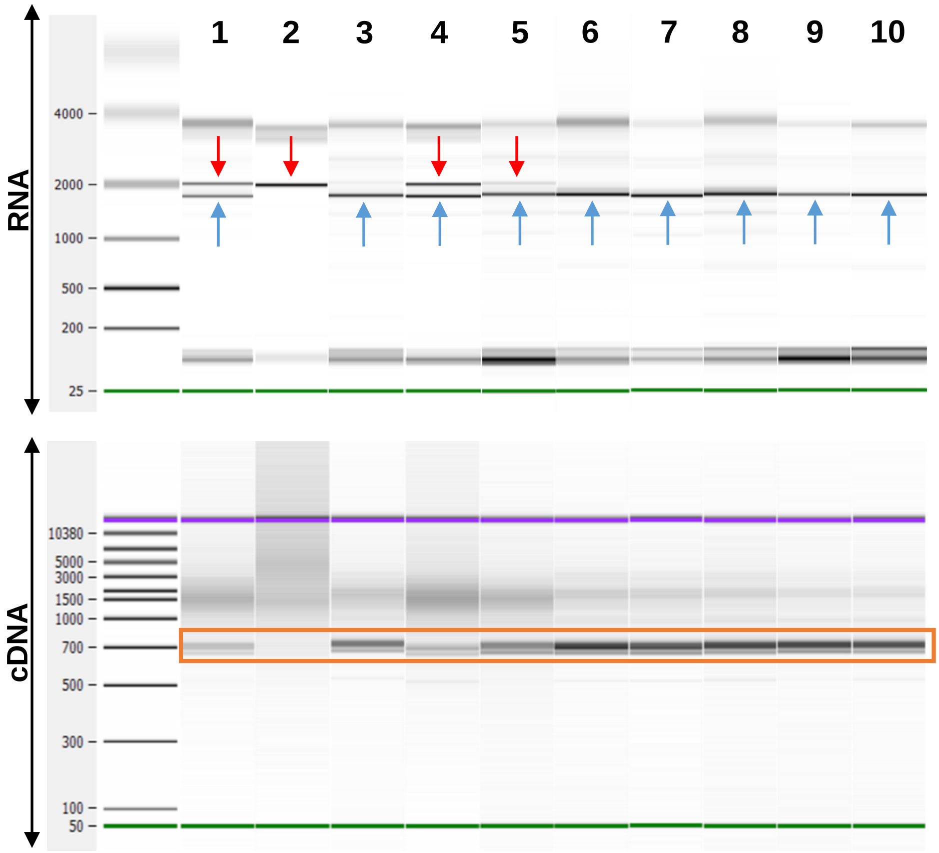

Supplementary Fig S9
Top panel: Agilent Bioanalyzer RNA gel images of TRAC2 field samples extracted with Direct-zol method with chloroform supplementation. Equal amounts of total RNA (50ng) were used for analysis. Samples were sorted by decreasing parasitemia. Two distinct 18S ribosomal bands can be seen in samples with medium parasitemias and in those with high parasitemia and high human RNA contamination. Red arrows indicate *P. falciparum* 18S rRNA; blue arrows indicate human 18S rRNA. In low parasitemia samples parasite's 18S band disappears completely below the detection limit (samples 6-10).
Bottom panel: Agilent Bioanalyzer cDNA traces amplified from RNA presented in the top panel. Equal amounts of amplified cDNA (20ng) were used for analysis. Orange box (~700 nt) indicates amplified human haemoglobin transcripts (HBA, HBB). With decreasing parasitemia haemoglobin bands intensify, with exception of the sample number 3 where despite high parasitemia an intensive haemoglobin transcripts bands can be seen presumably due to excessive human material presence.
Purple and green bands seen on the gels are upper and lower size markers respectively

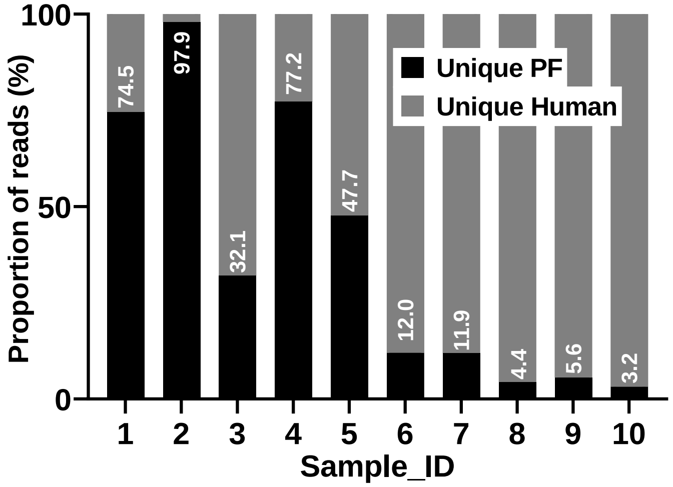

Supplementary Fig S10.
Sequencing alignment showing proportion of unique *P. falciparum* reads (numbers (%) shown in white inside the bars) to unique human reads from selected field samples. Higher numbers are observed with higher parasitemia (e.g. 1,2,4,5), or older parasites (e.g. 2). Low proportion of unique *P. falciparum* reads can be seen in sample no. 3 despite high parasitemia possibly due to incomplete depletion of WBC resulting in high human material presence.

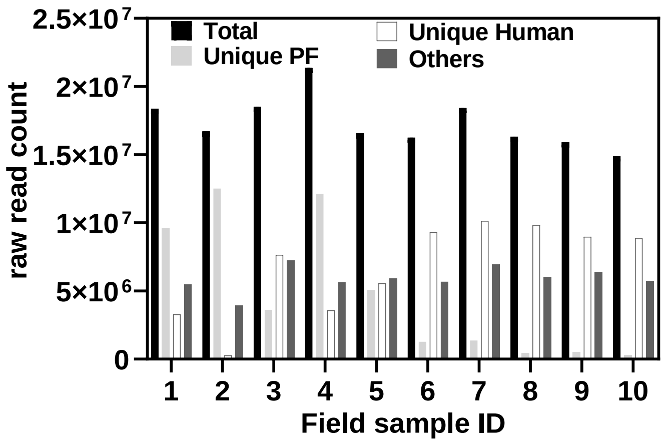

Supplementary Fig S11.
Raw read statistics from selected filed samples. Samples have been sorted by decreasing parasitemia. Total 24 libraries have been multiplexed per each lane on Illumina HiSeq4000 platform (final output 110Gb/lane).

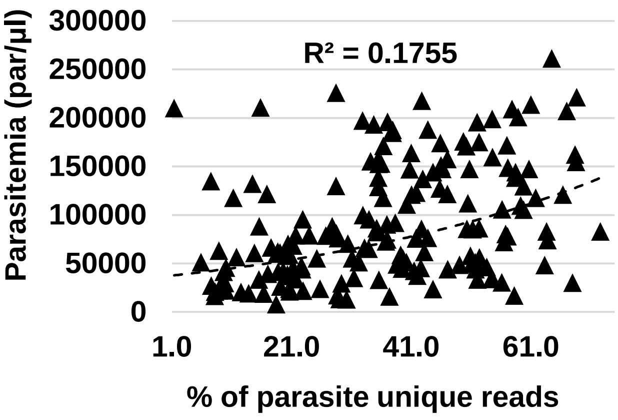

Supplementary Fig S12.
Correlation between the proportion of *P. falciparum* unique reads and parasitemia in 171 sequenced TRAC2 field samples.

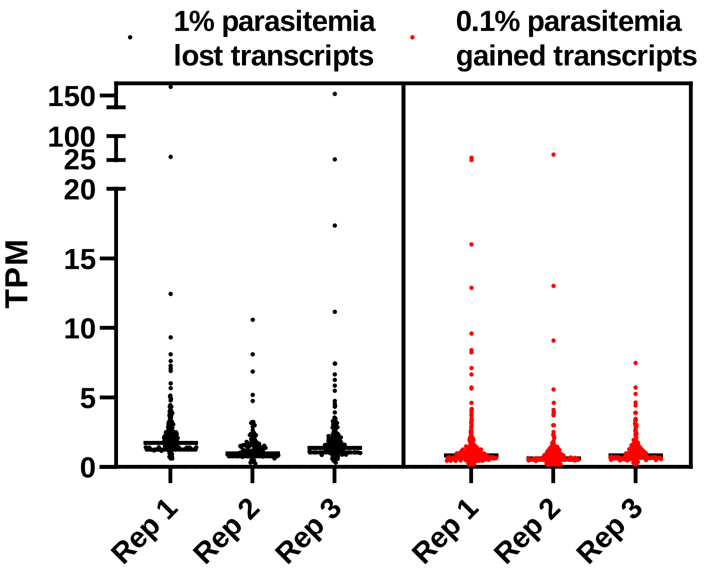

Supplementary Fig S13.
Expression values (shown as transcripts per million or TPM) of transcripts lost after gRNA-Cas9 treatment in high parasitemia samples (1%, black) and newly detected transcripts in low parasitemia samples (0.1%, red). Averaged values from three biological replicates are shown (per gene).
